# Revision of the Chinese *Cleptes* (Hymenoptera, Chrysididae) with description of new species

**DOI:** 10.3897/zookeys.362.6175

**Published:** 2013-12-13

**Authors:** Na-sen Wei, Paolo Rosa, Zai-fu Xu

**Affiliations:** 1 Department of Entomology, College of Natural Resources and Environment, South China Agricultural University, Guangzhou 510640, P. R. China; 2 Via Belvedere 8/d, I-20881 Bernareggio (MB), Italy

**Keywords:** Chrysididae, Cleptinae, revision, *Cleptes*, new species, China

## Abstract

The genus *Cleptes* Latreille, 1802 from China is revised and illustrated for the first time. Seventeen species of *Cleptes* are recorded. Nine species are new to science, *Cleptes albonotatus* **sp. n.**, *Cleptes eburnecoxis* **sp. n.**, *Cleptes flavolineatus* **sp. n.**, *Cleptes helanshanus* **sp. n.**, *Cleptes niger* **sp. n.**, *Cleptes shengi* **sp. n.**, *Cleptes sinensis* **sp. n.**, *Cleptes tibetensis* **sp. n.**, and *Cleptes villosus* **sp. n.**,and two species are reported as new to China, *Cleptes metallicorpus* Ha, Lee & Kim, 2011, and *Cleptes seoulensis* Tsuneki, 1959.

## Introduction

The small subfamily Cleptinae is considered to be the most plesiotypic group within Chrysididae. Its members are parasitoids of the prepupae of sawflies (Hymenoptera, Symphyta) in the families Tenthredinidae and Diprionidae (Tsuneki 1982; Kimsey and Bohart 1991; Móczár 1998b; Sheng et al. 1998; Wang et al. 2000).

Cleptinae includes three genera, *Cleptes* Latreille, 1802, *Cleptidea* Mocsáry, 1904, and *Lustrinia* Kurian, 1955, with 111 valid species (Kimsey and Bohart 1991; Móczár 1996a, 1997a, 1997b, 1998a, 1998b, 2000a, 2000b, 2001, 2009; Rosa 2003; Ha et al. 2011). Up to now only the genus *Cleptes* has been recorded from China.

The genus *Cleptes* can be distinguished from *Cleptidea* and *Lustrinia* by head as wide as long, eyes small and not bulging in frontal view, and claws with one small perpendicular submedial tooth.

*Cleptes* is mainly a Holarctic genus (Kimsey and Bohart 1991), with 90 recognized species (Kimsey and Bohart 1991; Móczár 1996a, 1996b, 1997a, 1997b, 1998a, 1998b, 1998c, 2000a, 2000b, 2001, 2009; Rosa 2003; Ha et al. 2011). Before this study, eight species were considered to be known from China (Table 1), of these, six are valid and two are misidentified.

Relatively thorough studies on *Cleptes* have been carried out in Europe and North America, compared with only few and non-systematic studies in Asia: Japan (Tsuneki 1959, 1982), Korea (Kim 1970; Kim and Ha 2009; Ha et al. 2011), Thailand (Tsuneki 1982), and China (Hammer 1950; Móczár 1968; Tsuneki 1982; Kimsey 1987; Rosa 2003). In China, the fauna of *Cleptes* is still poorly known. In this study, seventeen species of *Cleptes* are recognized, with nine of these are new to science, and two new to China.

**Table 1.** List of the Chinese species of Cleptinae before this study.

Species	Distribution
*Cleptes asianus* Kimsey, 1987	Taiwan (Oriental)
*Cleptes mandsuricus* Móczár, 1968	Northeast China (=Mandchuria) (Palaearctic)
*Cleptes mareki* Rosa, 2003	Shanxi (Palaearctic)
*Cleptes nitidulus* (Fabricius, 1793)	Northeast China (=Mandchuria) (Palaearctic) [misidentification]
*Cleptes semiauratus* (Linnaeus, 1761)	Jilin (Palaearctic) [misidentification]
*Cleptes sjostedti* Hammer, 1950	Jiangsu (=Kiangsu) (Palaearctic), Taiwan (Oriental)
*Cleptes taiwanus* Tsuneki, 1982	Taiwan (Oriental)
*Cleptes townesi* Kimsey, 1987	Taiwan (Oriental)

## Materials and methods

All the specimens were examined and described under a stereomicroscope (Olympus SZ61). All the photos were taken with a digital camera (CoolSNAP) attached to a Zeiss Stemi 2000-CS stereomicroscope. Images were processed using Image-Pro Plus software.

Morphological terminology of this study mainly follows that of Kimsey and Bohart (1991), Móczár (1998b), and Tsuneki (1959).

The abbreviations used in the descriptions are as follows: ASD= antennal socket diameter; F-I, F-II, F-III, etc. = flagellum I, flagellum II, flagellum III and so on; HH= head height, the maximum distance from the lowest margin of clypeus to the uppermost margin of frons in frontal view; HL= head length, the maximum distance across the head in lateral view; HW= head width, the maximum distance between compound eyes in frontal view; L/W = relative length to width; MOD= midocellar diameter; MS= malar space, the shortest distance between the base of mandibles and margin of the compound eyes; OCL= ocellar-occiputal line, the shortest distance between posterior ocellus and occipital carina; OOL= oculo-ocellar line, the shortest distance between posterior ocellus and compound eye; PD= puncture diameter; Ped= pedicel; POL= posterior ocellar line, the shortest distance between posterior ocelli; T-I, T-II, T-III, etc. = metasomal tergum I, tergum II, tergum III and so on.

Types and other specimens from the following institutions and private collections have been examined:

GA Gian Luca Agnoli private collection, Bologna, Italy.

MCNM Museo Civico di Storia Naturale, Milan, Italy.

MP Maurizio Pavesi private collection, Milan, Italy.

NHRS Swedish Museum of Natural History, Stockholm, Sweden.

NMW Naturhistorisches Museum, Zoologische Abteilung, Vienna, Austria.

PRC Paolo Rosa private collection, Bernareggio, Italy.

SCAU Hymenopteran Collection, South China Agricultural University, Guangzhou, China.

SHEM Shanghai Entomological Museum, Chinese Academy of Science, Shanghai, China.

ZJUH Parasitic Hymenoptera Collection of Zhejiang University, Hangzhou, China.

## Systematics

### Genus Cleptes

Latreille, 1802

http://species-id.net/wiki/Cleptes

Cleptes Latreille, 1802: 316. Type species: *Sphex semiaurata*Linnaeus 1761. Latreille 1802: 316; Aaron 1885: 211; Linsenmaier 1959: 7; Tsuneki 1959: 1; Móczár 1962: 115; Kimsey 1981: 801; Bohart and Kimsey 1982: 3; Kimsey and Bohart 1991: 43; Móczár 1996a: 153; 1996b: 134; 1997b: 26; 1998b: 502; 2000a: 319; 2000b: 297; 2001: 905; Rosa 2006: 82; Móczár 2009: 131; Ha et al. 2011: 491.

#### Diagnosis.

*Cleptes* can be distinguished from all other genera of Cleptinae by metasoma convex beneath, four visible tergites in females and five in males.

Other distinctive characteristics are: face convex; eyes not bulging and following the head profile in frontal view; malar space usually longer than 1 MOD; mandible robust, with two or more subapical teeth; clypeus usually emarginated beneath the antennal socket; pronotum narrowed anteriorly, and divided by a transverse crenate sulcus which delineates a bulbous collar; mesopleuron with subalar fossa and scrobal pit, scrobal sulcus and omaulus occasionally present; propodeum with long dorsal surface and vertical posterior declivity, posterolaterally angulate to dentate; claws with one small perpendicular submedial tooth; T-I and T-II dorsally subequal to or shorter than T-III and T-IV; forewing with weakly defined discoidal cell and an incomplete, or lacking, radial sector vein; ovipositor long and robust.

#### Distribution.

There are 90 valid *Cleptes* species in the world, 83 of which are found in the Holarctic region, eight in the Oriental region (two of which are in both the Holarctic and Oriental regions), and one in the Neotropical region.

#### Remarks.

Móczár (1962) and Kimsey (1981) divided the genus *Cleptes* into subgenera, which later were downgraded by Kimsey and Bohart (1991) into species groups. Recently Móczár (1997a, 1997b, 1998a, 1998b, 1998c, 2000a, 2000b, 2001) reviewed the genus by studying all the available types, and adopted the subgeneric and species group system. In this study we consider *Cleptes* subdivided into species groups, without subgeneric distinctions.

#### Keys to the Chinese species of the genus *Cleptes* Latreille

Females. Unknown for *Cleptes eburnecoxis* sp. n., *Cleptes mandsuricus*, *Cleptes sinensis* sp. n., *Cleptes tibetensis* sp. n., *Cleptes townesi*,and *Cleptes villosus* sp. n. Males. Unknown for *Cleptes albonotatus* sp. n., *Cleptes asianus*, *Cleptes flavolineatus* sp. n., *Cleptes helanshanus* sp.n., *Cleptes metallicorpus*, *Cleptes niger* sp. n., *Cleptes shengi* sp. n., and *Cleptes taiwanus*.

**Table:** 

1	Female: metasoma with four segments	2
–	Male: metasoma with five segments	12
2	Mesopleuron with V-shape loop (Plates 7D, 12D)	3
–	Mesopleuron without V-shape loop (Plates 1D, 3D, 4D, 6D, 8D, 10D)	7
3	Pronotum with longitudinal median sulcus complete (Plate 9C) or incomplete (Plate 12C)	4
–	Pronotum without longitudinal median sulcus (Plate 7C)	5
4	Posterior pit row of pronotum without considerably larger median pits; longitudinal median sulcus complete	*Cleptes seoulensis* Tsuneki
–	Posterior pit row of pronotum with two median pits considerably larger than the others (Plate 12C); longitudinal median sulcus incomplete	*Cleptes sjostedti* Hammer
5	Posterior pit row of pronotum with round pits and two median pits considerably larger than the others	*Cleptes asianus* Kimsey
–	Posterior pit row of pronotum with elongated pits and two median pits not considerably larger than the others (Plate 7C)	6
6	Mandibles with distinct striatopunctures; metanotum with two foveae along the posterior margin (Plate 7E)	*Cleptes metallicorpus* Ha, Lee & Kim
–	Mandibles without striatopunctures; metanotum with a broad fovea along the posterior margin	*Cleptes taiwanus* Tsuneki
7	Body without metallic colouration (Plates 1A, 3A, 8A)	8
–	Body with metallic colouration (Plates 4A, 6A, 10D)	10
8	Antennae with pedicels and F-I–F-III testaceous (Plate 3B) and rest of flagellum black; vertex with two oblique yellow stripes; mesosoma mostly black, with yellow stripes on pronotum and mesoscutellum (Plates 3C, 3E)	*Cleptes flavolineatus* sp. n.
–	Antennae with pedicels, F-I–F-III black (Plate 1B) or F-I blackish-brown and rest of flagellum black (Plate 8B); vertex and mesosoma entirely black (Plates 1A, 8A)	9
9	Metapleuron polished and weakly striate (Plate 8D); dorsal surface of propodeum with six longitudinal ridges and numerous weak transverse wrinkles (Plate 8E); propodeal angles long and blunt (Plate 8E)	*Cleptes niger* sp. n.
–	Metapleuron transversely striate (Plate 1D); dorsal surface of propodeum with dense and irregular wrinkles (Plate 1E); propodeal angles short and blunt (Plate 1E)	*Cleptes albonotatus* sp. n.
10	Pronotum with distinct posterior pit row (Plate 10C); metanotum with a big anteromedian pit and two foveae along the posterior margin (Plate 10E)	*Cleptes shengi* sp. n.
–	Pronotum without posterior pit row (Plates 4C, 6C); metanotum without anteromedian pit and fovea along the posterior margin (Plates 4E, 6C)	11
11	Pronotum golden, with green tints on lateral sides (Plate 4A); mesopleuron and metanotum golden-red (Plate 4E); mesopleuron somewhat polished, scrobal sulcus well defined (Plate 4D)	*Cleptes helanshanus* sp. n.
–	Pronotum and mesopleuron metallic blue, metanotum dark metallic blue (Plate 6A); mesopleuron with aligned and elongated punctures, scrobal sulcus not well defined (Plate 6D)	*Cleptes mareki* Rosa
12	Body entirely metallic green to blue; mesopleuron with V-shape loop complete (Plates 13D, 14D, 16D), or incomplete, missing of upper branch, not reaching anterior corner (Plate 9D)	13
–	Body not entirely metallic, only head and part of mesosoma with metallic colouration; mesopleuron without V-shape loop (Plates 2D, 11D, 15D)	16
13	Pronotum with complete or incomplete longitudinal median sulcus (Plates 9C, 13C); metanotum with a big anteromedian pit (Plates 9E, 13E)	14
–	Pronotum without longitudinal median sulcus (Plates 14C, 16C); metanotum with an indistinct or small anteromedian pit (Plates 14E, 16E)	15
14	Pronotum with longitudinal median sulcus complete (Plate 9C); posterior pit row of pronotum without considerably larger median pit (Plate 9C); mesopleuron with V-shape loop incomplete, missing of upper branch, not reaching anterior corner (Plate 9D)	*Cleptes seoulensis* Tsuneki
–	Pronotum with longitudinal median sulcus incomplete (Plate 13C); posterior pit row of pronotum with two median pits considerably larger than the others (Plate 13C); mesopleuron with complete and strong V-shape loop (Plate 13D)	*Cleptes sjostedti* Hammer
15	Face with sparse punctures (1.0–5.0 PD) (Plate 14B); frontal sulcus absent (Plate 14B); lower margin of clypeus with acute teeth at corners; head with sparse hairs on clypeus and vertex (Plate 14B); metanotum with transverse depression anteriorly and a broad fovea along the posterior margin (Plate 14E)	*Cleptes tibetensis* sp. n.
–	Face with dense punctures (0.5–1.0 PD) (Plate 16B); frontal sulcus complete but weak (Plate 16B); lower margin of clypeus without acute teeth at corners; head with dense hairs on clypeus and vertex (Plate 16B); metanotum without transverse impression anteriorly, with two medially fused foveae along the posterior margin (Plate 16E)	*Cleptes villosus* sp. n.
16	Pronotum with complete and narrow longitudinal median sulcus	*Cleptes mandsuricus* Móczár
–	Pronotum without longitudinal median sulcus (Plates 2C, 5D, 11C, 15C)	17
17	Body mostly blackish, with metallic colour restricted to face (Plate 15B) or head (Plate 2B)	18
–	Body with metallic blue colour on head and mesosoma (Plates 5A, 11A)	19
18	Face with metallic colour (Plate 15B); face and vertex with small and sparse punctures; dorsal surface of propodeum irregularly reticulate (Plate 15E); propodeal angles short and stumpy (Plate 15E)	*Cleptes townesi* Kimsey
–	Head entirely with metallic colour (Plates 2B, 2C); face with deep and dense punctures; dorsal surface of propodeum with six longitudinal ridges, with numerous and weak transverse wrinkles (Plate 2E); propodeal angles long and blunt (Plate 2E)	*Cleptes eburnecoxis* sp. n.
19	Metanotum with a broad fovea along the posterior margin (Plate 11E); metapleuron smooth and polished (Plate 11D)	*Cleptes sinensis* sp. n.
–	Metanotum without fovea along the posterior margin (Plate 5D); metapleuron transversally striate in upper part	*Cleptes mareki* Rosa

### Cleptes asianus

Kimsey, 1987

http://species-id.net/wiki/Cleptes_asianus

Cleptes asianus Kimsey, 1987: 56; Kimsey and Bohart 1991: 59; Móczár 2000a: 325.

#### Material examined.

None.

#### Diagnosis.

Body entirely purple, including femora and tibiae. Flagellum dark brown to black. Tegulae and tarsi brown. Lower margin of clypeus truncate. Dorsal surface of propodeum coarsely punctuate, with propodeal angles obtuse (Kimsey 1987; Móczár 2000a).

#### Distribution.

Oriental part of China (Taiwan).

#### Biology.

Collected in May.

#### Remarks.

*Cleptes asianus* Kimsey belongs to the *asianus* species-group (Móczár 2000a).

### Cleptes albonotatus sp. n.

http://zoobank.org/0481B833-7702-471B-8575-66D66C4570C2

http://species-id.net/wiki/Cleptes_albonotatus

Plate 1

#### Material examined.

Holotype ♀ (SCAU), Guangdong, Nanling National Nature Reserve (24°55'43"N, 113°1'1"E), 10–14.V.2006, Zai-fu Xu leg., No. SCAU-C0001.

#### Diagnosis.

*Cleptes albonotatus* sp. n. is similar to *Cleptes satoi* Tosawa based on the blackish colour of the body, coarse punctures on head, and irregular punctures along the posterior margin of pronotum; it resembles *Cleptes japonicus* Tosawa based on the transversely punctuate mesopleuron. However, *Cleptes albonotatus* sp. n. can be distinguished from these two species and others of the *satoi* species-group (*Cleptes flavolineatus* sp. n. and *Cleptes niger* sp. n.) by the combination of the following characteristics: body mostly blackish, without metallic reflections, face with close and coarse punctures, pronotum with indistinct posterior pit row, metanotum without anteromedian pit and pale tints on lateral sides of T-II.

#### Description.

**Plate 1.**
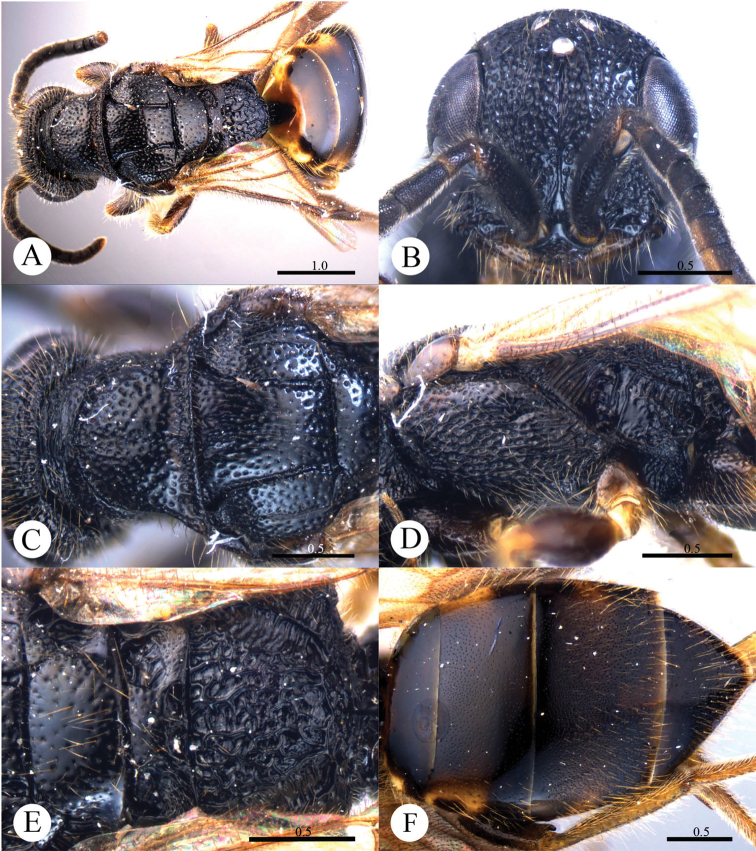
*Cleptes albonotatus* sp. n., holotype, female. **A** Habitus dorsal **B** Head anterior **C** Pronotum and mesoscutum dorsal **D** Mesopleuron and metapleuron lateral **E** Mesoscutellum, metanotum and propodeum dorsal **F** Metasoma dorsal. Scale bars in mm.

*Female*. Holotype. Body length 6.1 mm (Plate 1A). Forewing length 4.1 mm. HW: HH: HL = 37: 25: 50. POL: OOL: OCL = 8: 17: 18. MS = 1 MOD. Width of clypeal lower margin = 1.4 ASD. L/W of Ped, F-I, F-II, and F-III are 1.8, 1.3, 1.0, and 0.9, respectively.

*Head*. Face, ocellar area, and vertex with big, deep, close and coarse punctures (0–0.5 PD). Clypeus with lower margin truncate, without acute teeth at corners. Frontal sulcus complete (Plate 1B). Mandibles mostly polished, with few fine punctures and with four teeth. Ocellar triangle isosceles, without post-ocellar sulcus.

*Mesosoma*. Pronotum with punctures slightly smaller and sparser than those on vertex. Pronotum with distinct anterior pit row and indistinct posterior pit row; with irregular punctures along the posterior margin (Plate 1C); without longitudinal median sulcus (Plate 1C). Mesonotum and mesoscutellum with punctures similar to pronotum; notauli complete; parapsidal lines incomplete, 2/3 length of notauli; admedian lines incomplete and indistinct, 1/5 length of notauli (Plate 1C); axillary trough longitudinally striate. Mesopleuron transversely punctate, with short and indistinct scrobal sulcus (Plate 1D). Metanotum without anteromedian pit, with two foveae along the posterior margin; axillary trough longitudinally striate (Plate 1E). Metapleuron transversely striate (Plate 1D). Dorsal surface of propodeum with dense and irregular wrinkles. Propodeal angles short and blunt, slightly divergent (Plate 1E).

*Metasoma*. T-I impunctate; T-II–T-IV with small punctures (Plate 1F). Punctures on T-III denser than those on T-II and T-IV.

*Pubescence*. Head on vertex and clypeus with long (2.0–2.5 MOD), sparse and whitish hairs. Metasoma laterally on T-I and T-II with short (1 MOD), sparse and white hairs; dorsally and laterally on T-III and T-IV with long (2.2 MOD) and dense hairs.

*Colouration*. Head and mesosoma black, without metallic reflections. Mandibles black, with anterior half testaceous. Antennae black, with ventral sides of F-IV to F-XI testaceous. Tegulae blackish-brown. Legs blackish-brown, with trochanters, tibiae and tarsi testaceous. Metasoma blackish-brown, T-I laterally and on the anterior half testaceous, T-II laterally with distinct pale torus.

*Male*.Unknown.

#### Distribution.

Oriental part of China (Guangdong).

#### Biology.

Collected in May.

#### Etymology.

The specific name is referring to the pale torus on lateral T-II.

#### Remarks.

According to Móczár (2000b), *Cleptes albonotatus* sp. n. belongs to the *satoi* species-group based on the typical irregular punctures along the posterior margin of pronotum and black body.

### Cleptes eburnecoxis sp. n.

http://zoobank.org/74601C23-7620-47B5-BA8F-DEDB6B54EEB6

http://species-id.net/wiki/Cleptes_eburnecoxis

Plate 2

#### Material examined.

Holotype ♂ (SCAU), Zhejiang, Mt. Tianmu, Xianrending (30°20'56"N, 119°26'03"E), 25–29.VII.2011, Hua-yan Chen & Cheng-yuan Jin leg., No. SCAU-C0022. Paratypes: 2 ♂ (SCAU), Zhejiang, Mt. Tianmu, Xianrending, 25–29.VII.2011, Hua-yan Chen & Cheng-yuan Jin leg., No. SCAU-C0023, C0024; 5 ♂ (SCAU), Zhejiang, Mt. Tianmu, Xianrending, 25–29.VII.2011, Hua-yan Chen leg., No. SCAU-C0025–C0029; 1 ♂ (SCAU), Zhejiang, Mt. Tianmu, Xianrending, 29.VII.2003, Qiong Wu leg., No. 20034557; 2 ♂ (SCAU), Zhejiang, Mt. Tianmu, Xianrending, 28.VII.2003, Xue-xin Chen leg., No. 20038501, 20038502; 1 ♂ (SCAU), Mt. Tianmu, Xianrending, 27.VII.1999, Ming-shui Zhao leg., No. 997013; 1 ♂ (SCAU), Mt. Tianmu, Xianrending, 9.VIII.1998, Ming-shui Zhao leg., No. 994206; 1 ♂ (SCAU), Mt. Tianmu, Xianrending, 16.VIII.1999, Xue-xin Chen leg., No. 997278; 1 ♂ (SCAU), Guangxi, Longsheng, Huaping National Nature Reserve (32°52'36.84"N, 106°36'13.17"E), 25–26.VI.1982, Jun-hua He leg., No. 823647.

#### Diagnosis.

*Cleptes eburnecoxis* sp. n. is closely related to *Cleptes townesi* Kimsey based on the similar colouration, short MS (0.4 MOD), absence of posterior pit row on pronotum, polished mesopleuron, and posteriorly emarginate T-V. However, it can be distinguished by head being entirely metallic green (the metallic colour is restricted to face in *Cleptes townesi*), dorsal surface of propodeum with six longitudinal ridges (irregularly reticulate in *Cleptes townesi*), propodeal angles long and blunt (short and stumpy in *Cleptes townesi*).

#### Description.

**Plate 2.**
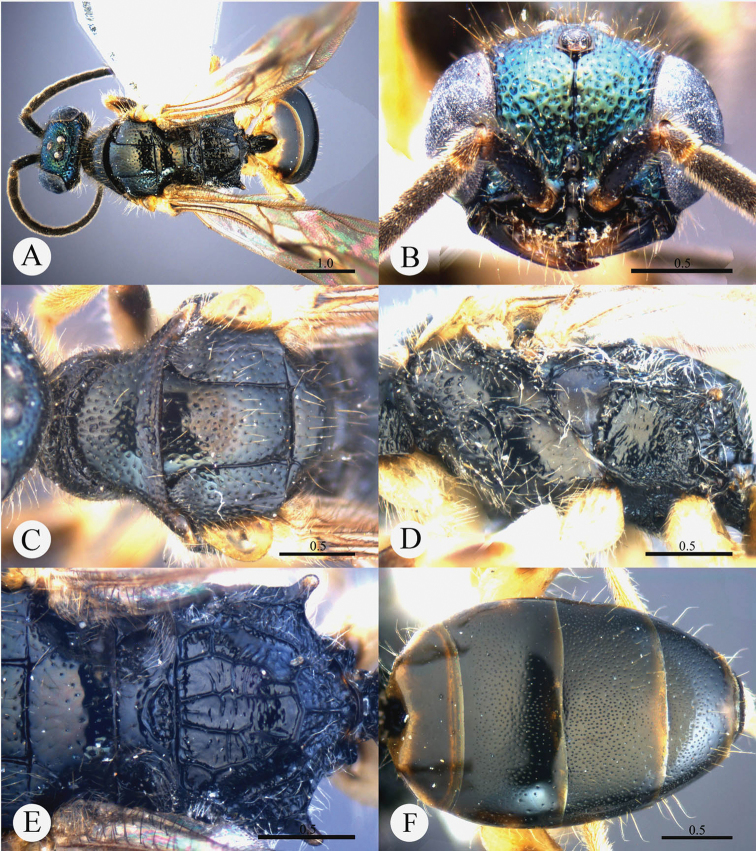
*Cleptes eburnecoxis* sp. n., holotype, male. **A** Habitus dorsal **B** Head anterior **C** Pronotum and mesoscutum dorsal **D** Mesopleuron and metapleuron lateral **E** Mesoscutellum, metanotum and propodeum dorsal **F** Metasoma dorsal. Scale bars in mm.

*Male*. Holotype. Body length 6.6 mm (Plate 2A). Forewing length 5.1 mm. HW: HH: HL = 36.5: 28: 17.5. POL: OOL: OCL = 8.5: 15: 19.5. MS = 0.4 MOD. Width of clypeal lower margin = 1.5 ASD. L/W of Ped, F-I, F-II, and F-III are 1.4, 2.4, 2.0, and 1.8, respectively.

*Head*. Face with deep and dense punctures (0–0.5 PD) on lateral sides, with shallow and sparse (0.5–1.0 PD) punctures medially. Clypeus with lower margin slightly convex medially, without acute teeth at corners. Frontal sulcus complete (Plate 2B). Mandibles with sparse punctures and three teeth. Ocellar area with dense punctures (0–0.5 PD). Ocellar triangle equilateral, with post-ocellar sulcus. Vertex with shallow and sparse punctures (0.5–1.0 PD).

*Mesosoma*. Pronotum with shallow and sparse punctures similar to those on vertex; with distinct anterior pit row, without posterior pit row (Plate 2C); without longitudinal median sulcus (Plate 2C). Mesonotum with small and shallow punctures, smaller and sparser on mesoscutellum; notauli complete; parapsidal lines incomplete, 3/4 length of notauli; admedian lines incomplete and indistinct, 1/4 length of notauli (Plate 2C); axillary trough polished, with few transverse wrinkles. Mesopleuron polished, with short scrobal sulcus, and with sparse punctures anteriorly (Plate 2D). Metanotum without anteromedian pit, with a big fovea along the posterior margin; axillary trough polished (Plate 2E). Metapleuron smooth and polished (Plate 2D). Dorsal surface of propodeum with six longitudinal ridges, with some weak transverse wrinkles. Propodeal angles long and blunt (Plate 2E).

*Metasoma*. T-I and T-V nearly impunctate; T-II–T-IV with small and dense punctures (Plate 2F). Punctures on T-III denser than those on T-II and T-IV. T-V with the posterior margin emarginate medially.

*Pubescence*. Head on clypeus, face, and vertex with long (1.5 MOD), sparse and brown hairs. Metasoma with few brown hairs scattered laterally. T-I and T-II laterally with short (0.8 MOD) hairs. T-III and T-IV laterally with long (1.5 MOD) hairs.

*Colouration*. Head entirely metallic green, rest of body without metallic reflections. Mandibles brown, with testaceous tints. Antennae blackish-brown. Mesosoma black, with basolateral angles of pronotum testaceous to white, with apex of propodeal angles brown. Tegulae testaceous. Legs with coxae, trochanters, and base of femora whitish; rest of femora, tibiae and tarsi testaceous. Metasoma blackish-brown, with anterior half of T-I, and lateral sides of T-I and T-II testaceous.

*Variation*. Body length 6.3–8.0 mm. Forewing length 4.3–5.5 mm. Head metallic greenish-blue or bluish-green. Clypeus with lower margin less convex than holotype, nearly truncate. Frontal sulcus complete, but indistinct on lower half. Pronotum black, with testaceous near the posterior margin. Admedian lines almost absent.

*Female*. Unknown.

#### Distribution.

Oriental part of China (Zhejiang, Guangxi).

#### Biology.

Collected from June to August.

#### Etymology.

The specific name refers to the whitish coxae.

#### Remarks.

According to Móczár (2000a), *Cleptes eburnecoxis* sp. n. would be the second member of the *townesi* species-group based on the short MS (0.4 MOD), blackish body and metallic tint on face.

### Cleptes flavolineatus sp. n.

http://zoobank.org/05E04406-5077-435B-9C39-010893A59209

http://species-id.net/wiki/Cleptes_flavolineatus

Plate 3

#### Material examined.

Holotype ♀ (ZJUH), Zhejiang, Hangzhou Botanical Garden (30°15'7.94"N, 120°7'40.48"E), 18.VI.1993, Jun-hua He leg., No.934793.

#### Diagnosis.

*Cleptes flavolineatus* sp. n. is similar to *Cleptes satoi* Tosawa, *Cleptes albonotatus* sp. n. and *Cleptes niger* sp. n. based on the blackish body, coarse punctures on head and rugose on pronotum. However, it can be easily distinguished by the combination of the following characteristics: with distinct yellow stripes on head, pronotum and mesoscutellum (absent in *Cleptes satoi*, *Cleptes albonotatus* sp. n. and *Cleptes niger* sp. n.); without anteromedian pit on metanotum (with an anteromedian pit in *Cleptes satoi*); with four longitudinal ridges and numerous transverse wrinkles dorsal surface of propodeum (propodeum coarsely striate in *Cleptes satoi*).

#### Description.

**Plate 3.**
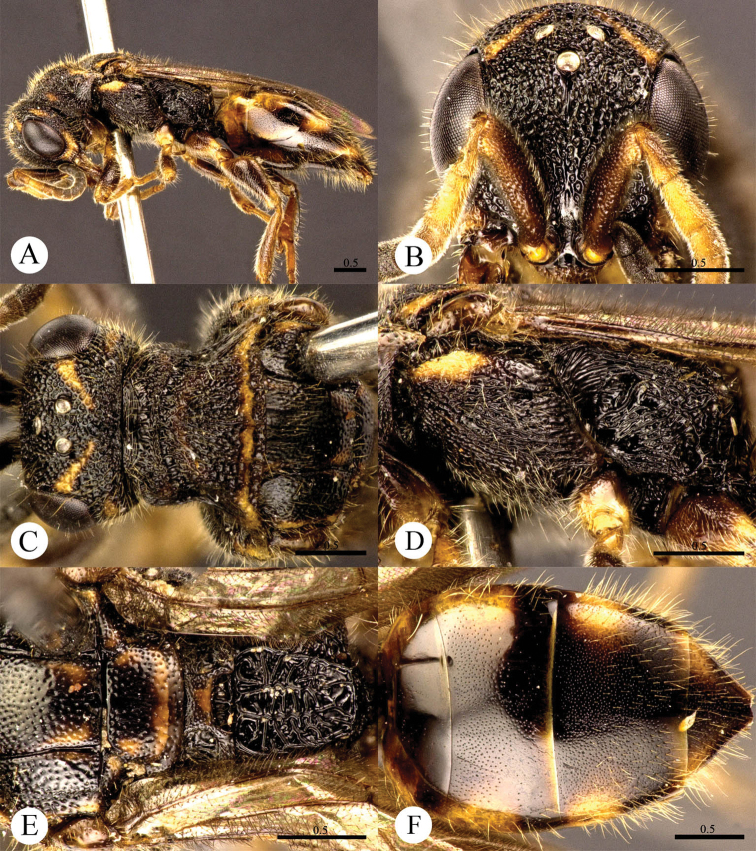
*Cleptes flavolineatus* sp. n., holotype, female. **A** Habitus lateral **B** Head anterior **C** Head, pronotum and mesoscutum dorsal **D** Mesopleuron and metapleuron lateral **E** Mesoscutum, mesoscutellum, metanotum and propodeum dorsal **F** Metasoma dorsal. Scale bars in mm.

*Female*. Holotype. Body length 5.7 mm (Plate 3A). Forewing length 4.1 mm. HW: HH: HL = 3.3: 2.9: 1.4. POL: OOL: OCL = 4: 10: 9. MS = 1.3 MOD. Width of clypeal lower margin = 1.4 ASD. L/W of Ped, F-I, F-II, and F-III are 1.9, 2, 1, and 0.78, respectively.

*Head*. Face with dense and coarse punctures (0–0.5 PD). Clypeus with lower margin indistinctly convex medially, without acute teeth at corners. Frontal sulcus incomplete, interrupted by coarse punctures (Plate 3B). Mandibles with few punctures and two teeth. Ocellar area with punctures similar to those on face. Ocellar triangle equilateral, without post-ocellar sulcus. Vertex with punctures similar to those on face.

*Mesosoma*. Pronotum rugose, with punctures merging in irregular lines. Pronotum with distinct anterior pit row and indistinct posterior pit row; with irregular punctures along the posterior margin (Plate 3C); without longitudinal median sulcus (Plate 3C). Mesonotum and metanotum with smaller, sparser and shallower punctures than those on pronotum; notauli complete; parapsidal lines incomplete, 3/4 length of notauli; admedian lines incomplete, 1/4 length of notauli (Plate 3C). Mesopleuron with dense and coarse punctures and transversely striate, with short scrobal sulcus (Plate 3D). Mesoscutellum longitudinally polish in the middle; axillary trough irregularly reticulate (Plate 3E). Metanotum without anteromedian pit, with a broad fovea along the posterior margin; axillary trough longitudinally and weakly striate (Plate 3E). Metapleuron strongly striate (Plate 3D). Dorsal surface of propodeum with four longitudinal ridges, with numerous transverse wrinkles. Propodeal angles short and blunt (Plate 3E).

*Metasoma*. T-I and posterior half of T-II nearly impunctate. Anterior half of T-II–T-IV with dense punctures (Plate 3F).

*Pubescence*. Head with long (1–1.5 MOD) and brown hairs. Metasoma dorsally and laterally on T-III and T-IV with short (0.8–1.0 MOD) and brown hairs.

*Colouration*. Head black, with two oblique yellow stripes dorsally. Mandibles brown. Antennae blackish-brown, with pedicels, F-I–F-III and ventral sides of F-IV–F-XI testaceous. Mesosoma mostly black, with a transverse yellow stripe near the posterior margin of pronotum, a transverse brown stripe on posterior mesoscutellum, and brown tints on mesoscutum between notauli and metanotum. Mesopleuron and metapleuron mostly black, with yellow tint on anterior corner of mesopleuron. Tegulae brown. Legs brown, with apical coxae and apical femora pale, tibiae and tarsi testaceous. Metasoma blackish-brown, with pale tint laterally on each segment.

*Male*. Unknown.

#### Distribution.

Oriental part of China (Zhejiang).

#### Biology.

Collected in June.

#### Etymology.

The specific name refers to the yellow stripes on head and pronotum.

#### Remarks.

According to Móczár (2000b), *Cleptes flavolineatus* sp. n. belongs to the *satoi* species-group based on the typical irregular punctures along the posterior margin of pronotum and black body.

### Cleptes helanshanus sp. n.

http://zoobank.org/B5329EF8-9EFA-4FBF-B74C-5ED9C60FCE79

http://species-id.net/wiki/Cleptes_helanshanus

Plate 4

#### Material examined.

Holotype ♀ (SCAU), Inner Mongolia, Mt. Helan (39°2'5.27"N, 106°1'38.09"E), 27.VII.2010, Hong-fei Chai leg., No. SCAU-C0002.

#### Diagnosis.

This species resembles *Cleptes mareki* Rosa based on the pronotum without posterior pit row and metanotum without pit or fovea. However, *Cleptes helanshanus* sp. n. can be quickly separated from this species by the colouration: pronotum golden, laterally with green tints, mesopleuron and metanotum golden red, and mesoscutellum greenish-golden.

#### Description.

**Plate 4.**
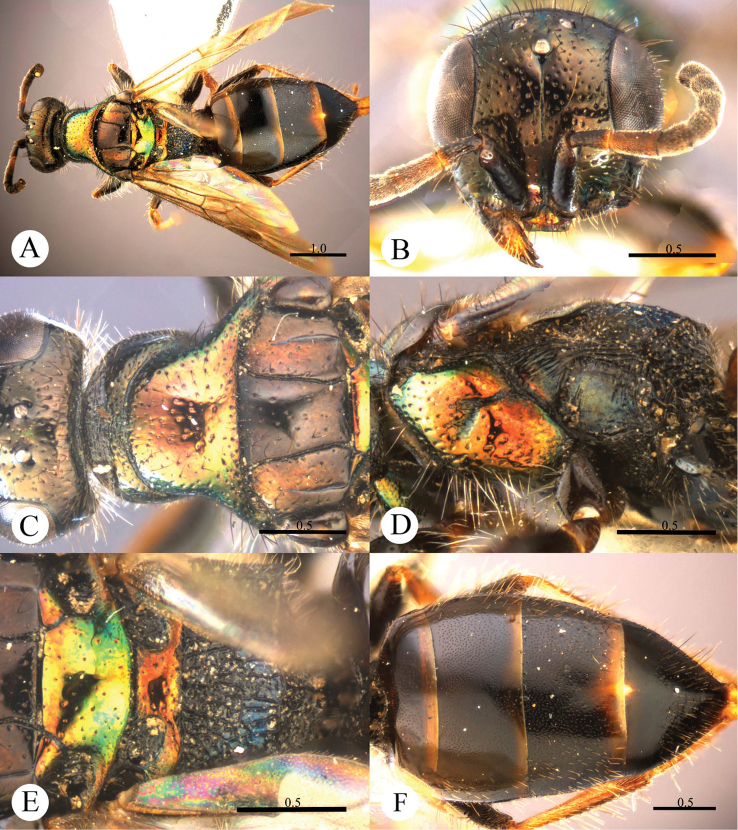
*Cleptes helanshanus* sp. n., holotype, female. **A** Habitus dorsal **B** Head anterior **C** Head, pronotum and mesoscutum dorsal **D** Mesopleuron and metapleuron lateral **E** Mesoscutellum, metanotum and propodeum dorsal **F** Metasoma dorsal. Scale bars in mm.

*Female*. Holotype. Body length 5.9 mm (Plate 4A). Forewing length 3.9 mm. HW: HH: HL = 79: 81: 38. POL: OOL: OCL = 10: 13: 18. MS = 1.7 MOD. Width of clypeal lower margin = 1.7 ASD. L/W of Ped, F-I, F-II, and F-III are 2.1, 2.1, 1.2, and 1.0, respectively.

*Head*. Face with small, shallow and sparse punctures (1–4 PD). Clypeus with lower margin truncate, without acute teeth at corners. Frontal sulcus complete, even if somewhat indistinct towards the clypeus (Plate 4B). Mandibles with sparse punctures and four teeth. Ocellar area and vertex with slightly denser punctures (1–3 PD). Ocellar triangle isosceles, without post-ocellar sulcus.

*Mesosoma*. Pronotum with small and sparse punctures similar to those on vertex; anterior pit row somewhat interrupted medially, without posterior pit row (Plate 4C); without longitudinal median sulcus (Plate 4C). Mesonotum and metanotum almost impunctate, with very sparse and shallow punctures. Mesoscutum with notauli complete; parapsidal lines incomplete, 3/4 length of notauli; admedian lines absent (Plate 4C); axillary trough indistinctly and longitudinally striate. Mesopleuron somewhat polished, with scrobal sulcus, and with sparse punctures on anterior half (Plate 4D). Transverse narrow sulcus present between mesoscutellum and metanotum (Plate 4E). Metanotum without pit or fovea; axillary trough weakly and irregularly reticulate (Plate 4E). Metapleuron polished, with some weak and transverse wrinkles on the upper part (Plate 4D). Dorsal surface of propodeum irregularly reticulate, with lateral margin straight and parallel. Propodeal angles short and stumpy (Plate 4E).

*Metasoma*. T-I with small and sparse punctures. T-II and T-III with dense punctures. T-IV with anterior half impunctate, and posterior half with sparse punctures (Plate 4F).

*Pubescence*. Head with long (2 MOD) and erect black bristles on vertex. Metasoma on T-III and T-IV dorsally and laterally with long (1.5 MOD) and white hairs; with very long (3–4 MOD) and erect black bristles laterally on T-III and T-IV.

*Colouration*. Head black, without metallic reflections. Mandibles blackish-brown, with teeth testaceous. Antennae black, with testaceous between pedicel and F-I. Pronotum golden, with green tints on lateral sides. Mesopleuron and metanotum golden-red. Mesoscutum black. Mesoscutellum greenish-golden. Metapleuron, propodeum and tegulae black. Legs blackish-brown, with tibiae and tarsi testaceous. Metasoma blackish-brown, with posterior margins of all segments testaceous, with small pale spot present between T-III and T-IV.

*Male*. Unknown.

#### Distribution.

Palaearctic part of China (Inner Mongolia).

#### Biology.

Collected in July.

#### Etymology.

The species is named after the type locality.

#### Remarks.

According to Móczár (1997b), *Cleptes helanshanus* sp. n. belongs to the *nitidulus* species-group based on the pronotum without posterior pit row and longitudinal median sulcus, and the blackish-brown metasoma.

### Cleptes mandsuricus

Móczár, 1968

http://species-id.net/wiki/Cleptes_mandsuricus

Cleptes mandsuricus Móczár, 1968: 171; Kimsey and Bohart 1991: 61; Móczár 1998a: 337.

#### Material examined.

None.

#### Diagnosis.

Head and mesosoma metallic bluish-green, with violet reflection. Tibiae metallic bluish, with violet reflection, tarsi brown. Pronotum with both anterior and posterior pit rows. Metanotum with an anteromedian pit and a broad fovea along the posterior margin (Móczár 1968, 1998a).

#### Distribution.

Palaearctic part of China (Northeast China).

#### Biology.

Collected in June.

#### Remarks.

*Cleptes mandsuricus* Móczár belongs to the *aerosus* species-group (Móczár 1998a).

### Cleptes mareki

Rosa, 2003

http://species-id.net/wiki/Cleptes_mareki

Plates 5 , 6

Cleptes mareki Rosa, 2003: 407.

#### Material examined.

Holotype ♂ (MCNM), “China c., 27.V., Zhongtiao Shan mt. c., 45 km W of Sanmenxia, Leg. J. Halada 1996”, “Holotypus, *Cleptes (Leiocleptes), mareki* n. sp., Paolo Rosa det. 2003”. Paratypes: 43 ♀+2 ♂ (PRC, GA and MP), “China c., 27.V., Zhongtiao Shan mt. c., 45 km W of Sanmenxia, Leg. J. Halada 1996”, “Paratypus, *Cleptes (Leiocleptes), mareki* n. sp., Paolo Rosa det. 2003”. Other material examined: 1 ♂ (SHEM), Gansu, Jiuquan, Huangnibao (39°43'0.60"N, 98°49'58.06"E), 1700 m, 15.VII. 2010, Xu-feng Zhang & Feng-li Cui leg.

#### Diagnosis.

**Plate 5.**
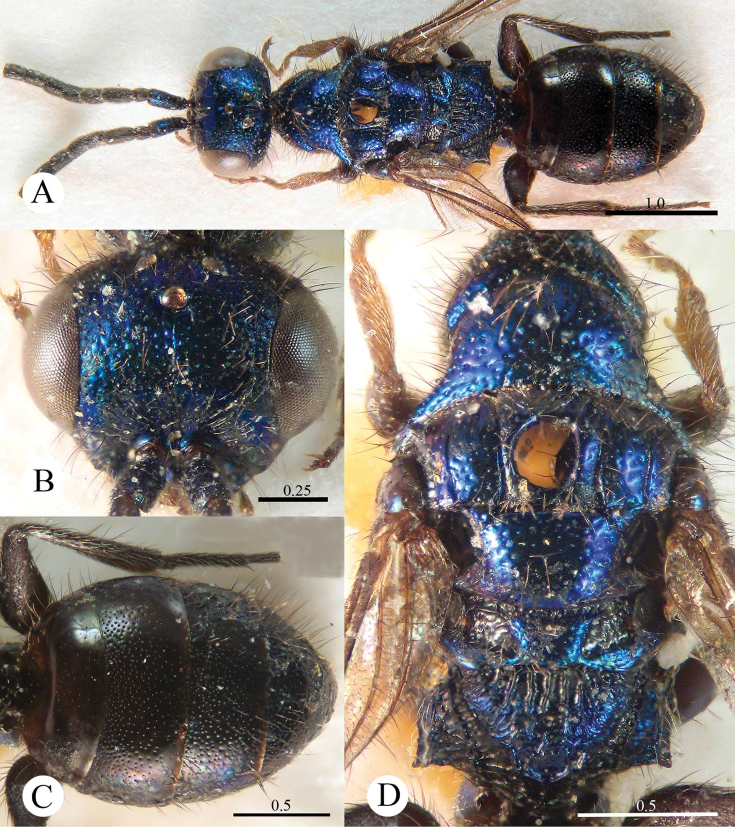
*Cleptes mareki* Rosa, 2003, holotype, male from Shanxi. **A** Habitus dorsal **B** Head anterior **C** Metasoma dorsal **D** Mesosoma dorsal. Scale bars in mm. (Photos by Michele Zilioli & Fabrizio Rigato).

**Plate 6.**
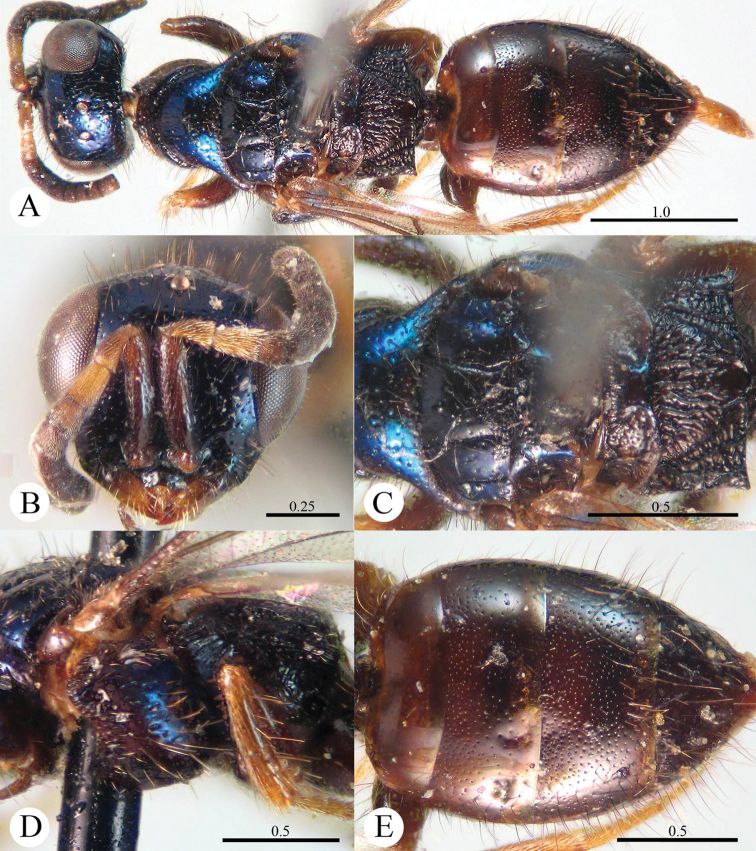
*Cleptes mareki* Rosa, 2003, paratype, female from Shanxi. **A** Habitus dorsal **B** Head anterior **C** Mesosoma dorsal **D** Mesopleuron and metapleuron lateral **E** Metasoma dorsal. Scale bars in mm. (Photos by Michele Zilioli & Fabrizio Rigato).

Addition to the original description. *Males*. Mesopleuron covered with sparse elongated punctures, almost polished towards metapleuron; without well-defined scrobal sulcus, but with enlarged fovea, more or less deeply excavated; metapleuron polished, transversally striate in upper part (Plate 5). *Females*. Punctuation on mesopleuron similar to the those of the males, with more aligned punctures; short scrobal sulcus, ending in a big and deep fovea; metapleuron entirely transversally striate (Plate 6).

*Variation*. The specimen from Gansu with head and mesosoma metallic greenish-blue.

#### Distribution.

Palaearctic part of China (Shanxi, Gansu).

#### Biology.

Collected in May and July at 1700 m.

#### Remarks.

*Cleptes mareki* Rosa belongs to the *nitidulus* species-group (Rosa 2003).

### Cleptes metallicorpus

**Plate 7.**
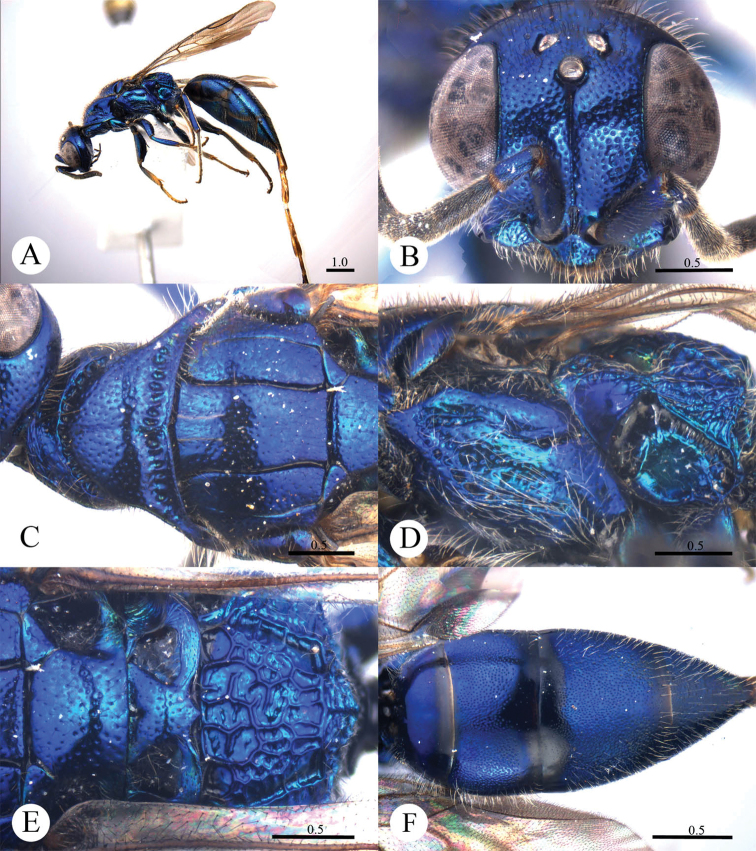
*Cleptes metallicorpus* Ha, Lee & Kim, 2011, female from Guangdong. **A** Habitus lateral **B** Head anterior **C** Pronotum and mesoscutum dorsal **D** Mesopleuron and metapleuron lateral **E** Mesoscutellum, metanotum and propodeum dorsal **F** Metasoma dorsal. Scale bars in mm.

Ha, Lee & Kim, 2011

http://species-id.net/wiki/Cleptes_metallicorpus

(New to China) Plate 7

Cleptes metallicorpus Ha, Lee & Kim,2011: 489.

#### Material examined.

1 ♀ (SCAU), Guangdong, Nanling National Nature Reserve (24°55'43"N, 113°1'1"E), 10–14.V.2006, Zai-fu Xu leg., No. SCAU-C0004; 1 ♀ (SCAU), Zhejiang, Mt. Tianmu, Xianrending (30°20'56"N, 119°26'03"E﻿﻿), 25–29.VII.2011, Hua-yan Chen & Cheng-yuan Jin leg., No. SCAU-C0005; 1 ♀ (ZJUH), Mt. Tianmu, Qiliting (30°20'N, 119°26'E), 13.VI.1998, Xue-xin Chen leg., No. 980875; 1 ♀ (PRC), Shaanxi, Quing Ling Shan mts, Road Baoji – Taibal vill pass, 40 km S Baoji Zd, 21–23.June.1998, Jindra lgt.

#### Diagnosis.

Frontal sulcus complete. Pronotum with distinct anterior and posterior pit rows, with elongated pits of posterior pit row. Mandibles with distinct striatopunctures. Mesopleuron with distinct V-shape loop. Metapleuron mostly smooth and polished.

#### Description.

Redescribed after a female from Guangdong. Body length 9.4 mm (Plate 7A). Forewing length 6.1 mm. HW: HH: HL = 22.8: 18.5: 13.8. POL: OOL: OCL = 6: 8: 16.8. MS = 1 MOD. Width of clypeal lower margin = 1.4 ASD. L/W of Ped, F-I, F-II, and F-III are 2, 3, 1.2, and 0.9, respectively.

*Head*. Face, ocellar area and vertex with small, shallow and sparse punctures (0.5–1.5 PD). Clypeus with lower margin truncate medially and concave laterally before short acute teeth at corners. Frontal sulcus complete (Plate 7B). Mandibles with distinct striatopunctures. Ocellar triangle isosceles, without post-ocellar sulcus.

*Mesosoma*. Pronotum with punctures similar to those on vertex. Pronotum with distinct anterior and posterior pit rows, with pits of posterior pit row elongated (Plate 7C); without longitudinal median sulcus (Plate 7C). Mesonotum and metanotum with smaller, shallower and sparser punctures than those on pronotum. Mesoscutum notauli complete; parapsidal lines nearly complete; admedian lines incomplete, 1/3 length of notauli (Plate 7C); axillary trough smooth. Mesopleuron with distinct V-shape loop (Plate 7D). Metanotum with a small anteromedian pit, with two foveae along the posterior margin; axillary trough smooth (Plate 7E). Metapleuron mostly smooth and polished (Plate 7D). Dorsal surface of propodeum irregularly reticulate; lateral margins parallel. Propodeal angles short and nearly right-angled (Plate 7E).

*Metasoma*. T-I nearly impunctate; T-II–T-IV with dense punctures (Plate 7F). Punctures on T-III denser than those on T-II and T-IV.

*Pubescence*. Head with erect, short (0.5–1.0 MOD), sparse and black bristles, but with short (1 MOD) and brown hairs on the posterior margin of vertex. Clypeus with sparse, long (2 MOD) and white hairs. Metasoma dorsally and laterally with long (1.0–1.5 MOD) and white hairs.

*Colouration*. Head and mesosoma metallic bluish-green with purple tints. Mandibles metallic greenish-blue, with teeth blackish-brown. Antennae blackish-brown, with scapes and pedicels metallic bluish-green. Legs metallic blue, with tarsi testaceous. Metasoma metallic bluish-purple, with black streak on segmental margins.

*Variation*. Body length 6.7–9.7 mm. Forewing length 4.5–6.4 mm. Face with small, shallow and slightly denser punctures (0.5–1.5 PD). Clypeus with less distinct acute teeth at corners of lower margin. Metasoma with some metallic green tints.

*Male*. Unknown.

#### Distribution.

China (Shaanxi, Zhejiang, Guangdong); Korea.

#### Biology.

Collected from May to July.

#### Remarks.

According to Móczár (2000a), *Cleptes metallicorpus* Ha et al. belongs to the *asianus* species-group based on two distinct pit rows on pronotum, longitudinal median sulcus absent and mesopleuron with V-shape loop.

### Cleptes niger sp. n.

http://zoobank.org/DF7C665C-7A5F-4112-AA88-1789CAC0CB59

http://species-id.net/wiki/Cleptes_niger

Plate 8

#### Material examined.

Holotype ♀ (SCAU), Shaanxi, Mt. Taibai (34°5'12.41"N, 107°42'41.77"E), 1100 m, 12–13.VII.2012, Na-sen Wei leg., No. SCAU-C0030. Paratype: 1 ♀ (SCAU), Shaanxi, Mt. Taibai (34°5'12.41"N, 107°42'41.77"E), 1100 m, 12–13.VII.2012, Na-sen Wei leg., No. SCAU-C0031.

#### Diagnosis.

*Cleptes niger* sp. n. is related to *Cleptes albonotatus* sp. n., *Cleptes flavolineatus* sp. n. and *Cleptes satoi* Tosawa based on the blackish body, coarse punctures on head, rugose pronotum and similar sculptures on the mesopleuron. However, it can be distinguished by the combination of the following characteristics: body without metallic tints or yellow stripes (mesopleuron partly metallic blue in *Cleptes satoi*, body with yellow stripes in *Cleptes flavolineatus* sp. n.); metanotum without anteromedian pit (with an anteromedian pit in *Cleptes satoi*); dorsal surface of propodeum with six longitudinal ridges and numerous and weak transverse wrinkles (irregularly reticulate with dense wrinkles in *Cleptes satoi* Tosawa and *Cleptes albonotatus* sp. n.,); metapleuron polished and weak striate on upper part (entirely and strongly striate in *Cleptes albonotatus* sp. n. and *Cleptes flavolineatus* sp. n.).

#### Description.

**Plate 8.**
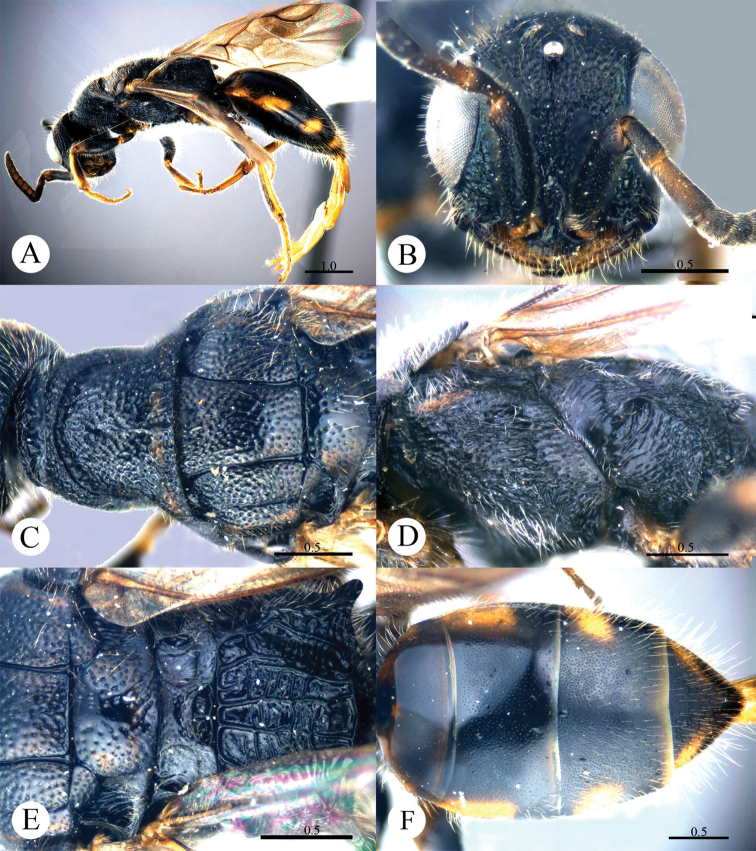
*Cleptes niger* sp. n., holotype, female. **A** Habitus lateral **B** Head anterior **C** Pronotum and mesoscutum dorsal **D** Mesopleuron and metapleuron lateral **E** Mesoscutellum, metanotum and propodeum dorsal **F** Metasoma dorsal. Scale bars in mm.

*Female*. Holotype. Body length 7.3 mm (Plate 8A). Forewing length 4.8 mm. HW: HH: HL = 14: 12.5: 8. POL: OOL: OCL = 9: 19: 23. MS = 1.2 MOD. Width of clypeal lower margin = 1.4 ASD. L/W of Ped, F-I, F-II, and F-III are 2.1, 2.4, 1.1, and 0.8, respectively.

*Head*. Face, ocellar area and vertex with dense and coarse punctures (0–0.5 PD). Frontal sulcus complete, indistinct on lower face (Plate 8B). Clypeus with lower margin convex medially, without acute teeth at corners. Mandibles with sparse punctures and three teeth. Ocellar triangle isosceles, with post-ocellar sulcus indistinct and curvate.

*Mesosoma*. Pronotum rugose, with coarse and dense punctures. Pronotum with a distinct anterior pit row, without posterior pit row and defined pits, but with shallow depression with irregular punctures along the posterior margin (Plate 8C); without longitudinal median sulcus; with a small depression in the middle of pronotum (Plate 8C). Mesonotum and metanotum with punctures smaller and sparser than on pronotum. Mesoscutum with notauli complete; parapsidal lines incomplete, 1/2 length of notauli; admedian lines absent (Plate 8C). Mesopleuron with dense and coarse punctures and transverse wrinkles; scrobal sulcus short (Plate 8D). Mesoscutellum longitudinally polish in the middle; axillary trough longitudinally striate (Plate 8E). Metanotum without anteromedian pit, with a broad fovea along the posterior margin; axillary trough mostly smooth, longitudinally and weakly striate (Plate 8E). Metapleuron polished and weakly striate on upper part (Plate 8D). Dorsal surface of propodeum with six longitudinal ridges, with numerous and weak transverse wrinkles. Propodeal angles long and blunt (Plate 8E).

*Metasoma*. T-I nearly impunctate. T-II–T-IV with dense punctures (Plate 8F).

*Pubescence*. Head with long (1.5–2.0 MOD) and white hairs. Metasoma on T-III and T-IV dorsally and laterally with very long (2.0–2.5 MOD) and brownish hairs.

*Colouration*. Head and mesosoma entirely black, without metallic reflections. Mandibles testaceous. Antennae blackish-brown, with ventral sides of F-IV to F-XI testaceous. Tegulae black. Legs with coxae and femora blackish-brown, tibiae and tarsi testaceous. Metasoma black, with T-I blackish-brown anteriorly, T-I to T-III with distinct testaceous tint laterally.

*Variation*. Paratype: Body length 7.1 mm. Forewing length 4.8 mm.

*Male*. Unknown.

#### Distribution.

China (Shaanxi).

#### Biology.

Collected in July at 1100 m.

#### Etymology.

The specific name refers to the black colouration.

#### Remarks.

According to Móczár(2000b), *Cleptes niger* sp. n. belongs to the *satoi* species-group based on the typical irregular punctures along the posterior margin of pronotum, and black body.

### Cleptes seoulensis

**Plate 9.**
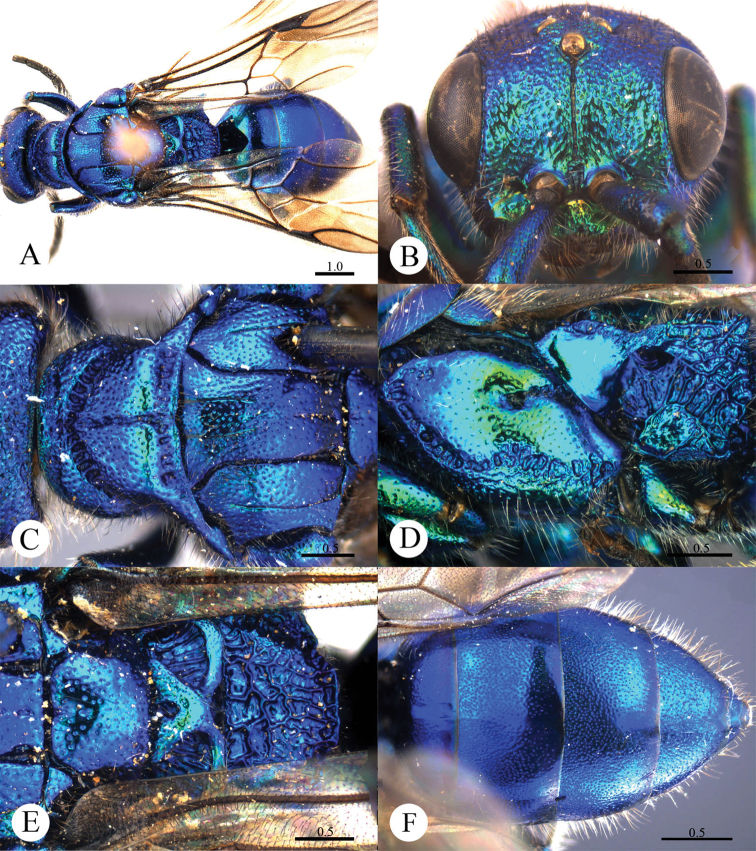
*Cleptes seoulensis* Tsuneki, 1959, male from Anhui. **A** Habitus dorsal **B** Head anterior **C** Pronotum and mesoscutum dorsal **D** Mesopleuron and metapleuron lateral **E** Mesoscutellum, metanotum and propodeum dorsal **F** Metasoma dorsal. Scale bars in mm.

Tsuneki, 1959

http://species-id.net/wiki/Cleptes_seoulensis

(New to China) Plate 9

Cleptes seoulensis Tsuneki, 1959: 13; Kim 1970: 506; Kimsey and Bohart 1991: 64; Móczár 1998a: 340; Ha et al. 2011: 492.

#### Material examined.

1 ♂ (SCAU), Anhui, Jinzhai, Tiantangzhai (31°8'19.23"N, 115°46'35.82"E), 1000 m, 2.VI.2006, Hu Zhou leg., No. SCAU-C0007.

#### Diagnosis.

Body mostly metallic greenish-blue, with some purple tints. Ocellar area yellowish-green. Pronotum with longitudinal median sulcus complete and foveate. Female with complete V-shape loop on mesopleuron. Male with incomplete V-shape loop, missing the upper branch, not reaching anterior corner. Metanotum with a big and oval anteromedian pit, with a fovea along the posterior margin. Metapleuron mostly smooth and polished.

#### Description.

Redescribed after a male from Anhui. Body length 10.0 mm (Plate 9A). Forewing length 7.5 mm. HW: HH: HL = 30.3: 24.3: 15. POL: OOL: OCL = 7: 12: 16. MS = 1.6 MOD. Width of clypeal lower margin = 1.3 ASD. L/W of Ped, F-I, F-II, and F-III are 1.2, 2, 1.4, and 1.5, respectively.

*Head*. Face and ocellar area with dense and coarse punctures (0–0.5 PD), punctures shallower on lower face. Frontal sulcus complete (Plate 9B). Clypeus with lower margin truncate, with shallow and sparse punctures (1 PD), without acute teeth at corners. Mandibles with sparse punctures and four teeth. Ocellar triangle isosceles, without post-ocellar sulcus. Vertex with punctures similar to those on face, but shallower.

*Mesosoma*. Pronotum with punctures smaller and sparser (0.5–1.0 PD) than those on vertex. Pronotum with distinct anterior and posterior pit rows (Plate 9C); with longitudinal median sulcus complete and foveate (Plate 9C). Mesonotum and metanotum with punctures gradually smaller and sparser than those on pronotum. Mesoscutum with notauli complete; parapsidal lines incomplete, 4/5 length of notauli; admedian lines incomplete, 2/5 length of notauli; indistinct longitudinal and slightly foveate sulcus present medially on posterior part of mesoscutum, 1/4 length of notauli (Plate 9C); axillary trough longitudinally striate. Mesopleuron with foveate V-shape loop incomplete, missing of upper branch, not reaching anterior corner (Plate 9D). Metanotum with a big and oval anteromedian pit, with a fovea along the posterior margin; axillary trough longitudinally striate (Plate 9E). Metapleuron mostly smooth and polished (Plate 9D). Dorsal surface of propodeum irregularly reticulate; lateral margins parallel, slightly concave before propodeal angles (Plate 9E).

*Metasoma*. T-I, T-IV and T-V with sparse punctures. T-II and T-III with dense punctures (Plate 9F).

*Pubescence*. Head with long (1–2 MOD) and brown hairs, except upper face, and vertex with short (0.8–1.0 MOD), erect and black bristles. Metasoma on T-III and T-IV dorsally and laterally with long (1.0–1.5 MOD), sparse and white hairs.

*Colouration*. Body mostly metallic greenish-blue, with some purple tints. Ocellar area yellowish-green. Mandibles metallic blue, with teeth blackish-brown. Antennae black, with scapes and pedicels metallic bluish-green. Tegulae metallic bluish-green. Legs metallic bluish-green, with tarsi testaceous.

*Female*. Not available specimens for this study.

#### Distribution.

China (Anhui); Korea.

#### Biology.

Collected in June at 1000 m.

#### Remarks.

*Cleptes seoulensis* Tsuneki belongs to the *fudzi* species-group (Móczár 1998a).

### Cleptes shengi sp. n.

http://zoobank.org/0D21B062-BAF9-43E0-9401-215500ADE0CD

http://species-id.net/wiki/Cleptes_shengi

Plate 10

Cleptes semiauratus : Sheng et al. 1998: 7 (misidentification).

#### Material examined.

Holotype ♀ (SCAU), Jilin, Maoershan National Forest Park (42°51'23.72"N, 129°28'12.36"E), 15.VI.2010, Mao-ling Sheng leg., No. SCAU-C0035.

#### Diagnosis.

*Cleptes shengi* sp. n. is related to *Cleptes semiauratus* (Linnaeus), based on the similar sculptures on pronotum and mesopleuron. However, it can be separated by the evident differences in colouration: head and mesosoma mostly black, mesopleuron with metallic blue (head and pronotum flame red, mesonotum and metanotum golden red or golden green in *Cleptes semiauratus*).

#### Description.

**Plate 10.**
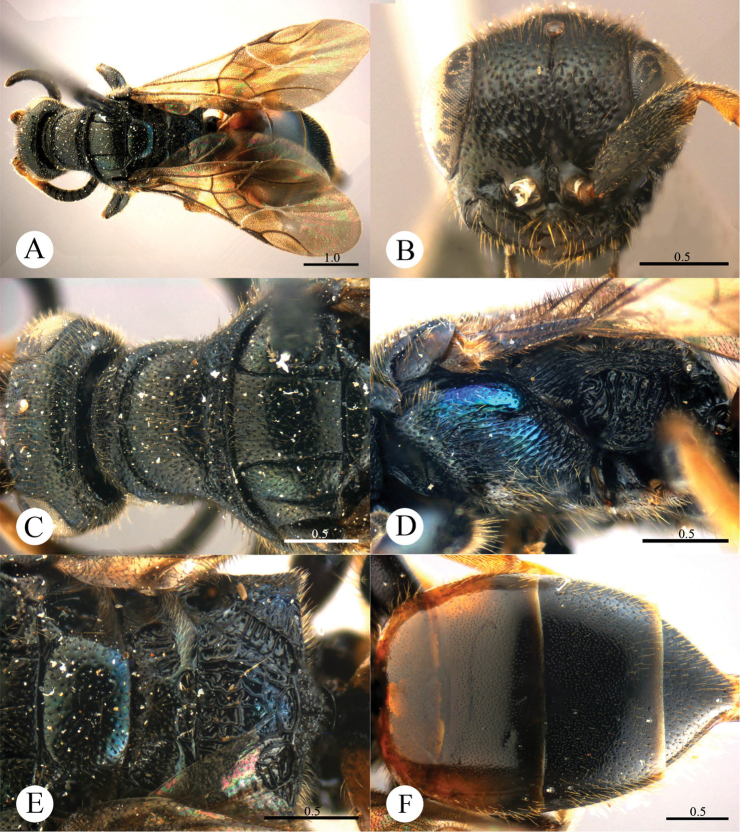
*Cleptes shengi* sp. n., holotype, female. **A** Habitus dorsal **B** Head anterior **C** Head, pronotum and mesoscutum dorsal **D** Mesopleuron and metapleuron lateral **E** Mesoscutellum, metanotum and propodeum dorsal **F** Metasoma dorsal. Scale bars in mm.

*Female*. Holotype.Body length 6.7 mm (Plate 10A). Forewing length 4.6 mm. HW: HH: HL = 15: 14: 8. POL: OOL: OCL = 4.8: 6: 8.8. MS = 1.4 MOD. Width of clypeal lower margin = 1.2 ASD. L/W of Ped, F-I, F-II, and F-III are 1.9, 2.0, 1.0, and 0.8, respectively.

*Head*. Head with dense punctures (1 PD), and slightly denser and coarser on vertex. Frontal sulcus incomplete, interrupted medially (Plate 10B). Clypeus with lower margin truncate, with indistinct acute teeth at corners. Mandible with three teeth. Ocellar triangle isosceles, without post-ocellar sulcus.

*Mesosoma*. Pronotum with similar punctures to those on vertex. Pronotum with a distinct anterior and posterior pit rows (Plate 10C); without longitudinal median sulcus (Plate 10C). Mesonotum with shallow and sparse punctures. Mesoscutum with notauli complete; parapsidal lines incomplete, 3/4 length of notauli; admedian lines absent; axillary trough with tubercle-like process (Plate 10C). Mesopleuron transversely striate, with short scrobal sulcus (Plate 10D). A foveate transverse sulcus present between mesoscutellum and metanotum (Plate 10E). Metanotum with a big and oval anteromedian pit, with two foveae along the posterior margin (Plate 10E). Metapleuron obliquely and strongly striate (Plate 10D). Dorsal surface of propodeum irregularly reticulate. Propodeal angles short and blunt (Plate 10E).

*Metasoma*. T-I and T-IV with sparse punctures. T-II and T-III with dense punctures (Plate 10F).

*Pubescence*. Face and vertex with short (0.8–1.0 MOD) and black hairs. Clypeus and mandibles with long (1.5–2.0 MOD), sparse and testaceous bristles. Metasoma on T-I and T-II laterally with very short (0.5 MOD), sparse and whitish hairs; on T-III and T-IV laterally, and on T-IV dorsally with long (1.0–1.3 MOD) hairs.

*Colouration*. Head, mandibles, scapes, mesosoma, tegulae, coxae, and femora black, with mesopleuron metallic blue. Antennae black, with pedicels, F-I, partly F-II testaceous. Legs black, with tibiae and tarsi testaceous. Metasoma black, with testaceous on T-I anteriorly and laterally and T-II laterally.

*Male*. Unknown.

#### Distribution.

Palaearctic part of China (Jilin).

#### Biology.

Parasitoids of*Pachynematus itoi* Okutani (Sheng et al. 1998). Collected in June.

#### Etymology.

The species is named after the collector.

#### Remarks.

According to Móczár (2001), *Cleptes shengi* sp. n. belongs to the *semiauratus* species-group based on the pronotum without longitudinal median sulcus and with both anterior and posterior pit rows distinct, and the colouration of metasoma.

### Cleptes sinensis sp. n.

http://zoobank.org/F84DCD2C-7730-49B3-AC88-766E36B3D5D5

http://species-id.net/wiki/Cleptes_sinensis

Plate 11

#### Material examined.

Holotype ♂ (SCAU), Shaanxi, Liping National Forest Park (32°51'43"N, 106°34'56"E), 23.VII.2004, Hong-ying Zhang leg., No.SCAU-C0010. Paratypes: 1 ♂ (SCAU), Shaanxi, Mt. Taibai (34°5'12.41"N, 107°42'41.77"E), 12–13.VII.2012, Na-sen Wei leg., No. SCAU-C0011; 6 ♂ (SCAU), Shaanxi, Liping National Forest Park (32°52'36.84"N, 106°36'13.17"E), 1344 m, 23.VII.2004, Qiong Wu leg., No. 20046895–20046900; 1 ♂ (SCAU), Shaanxi, Liuba, Mt. Zibo (33°39'24.42"N, 106°46'48.70"E), 1632 m, 4.VIII.2004, Xue-xin Chen leg., No. 20047129; 1 ♂ (SCAU), Hainan, Jianfengling National Nature Reserve (18°45'26.19"N, 108°53'6.75"E), 9.V.2008, Jing-xian Liu leg., No. 200800141; 1 ♂ (SCAU), Sichuan, Wolong National Nature Reserve (31°8'25.07"N, 103°8'36.32"E), 21.VII.2006, Hong-ying Zhang leg., No. 200610800; 1 ♂ (SCAU), Hubei, Wufeng, Houhe National Nature Reserve (30°4'40.64"N, 110°37'32.89"E), 11.VII.1999, Wen-jun Bu leg., No. 200104521.

#### Diagnosis.

*Cleptes sinensis* sp. n. shares the metallic blue colouration on head and part of mesosoma, pronotum without posterior pit row, and polished mesopleuron with *Cleptes nitidulus* (Fabricius) and *Cleptes mareki* Rosa. However, it can be distinguished by metasoma blackish-brown (T-I and T-II testaceous in *Cleptes nitidulus* and black with weak blue reflections in *Cleptes mareki*); metanotum with a broad fovea along the posterior margin (without broad fovea in *Cleptes nitidulus* and *Cleptes mareki*); dorsal surface of propodeum with six longitudinal ridges and numerous and weak transverse wrinkles (irregularly reticulate in *Cleptes nitidulus* and *Cleptes mareki*).

#### Description.

**Plate 11.**
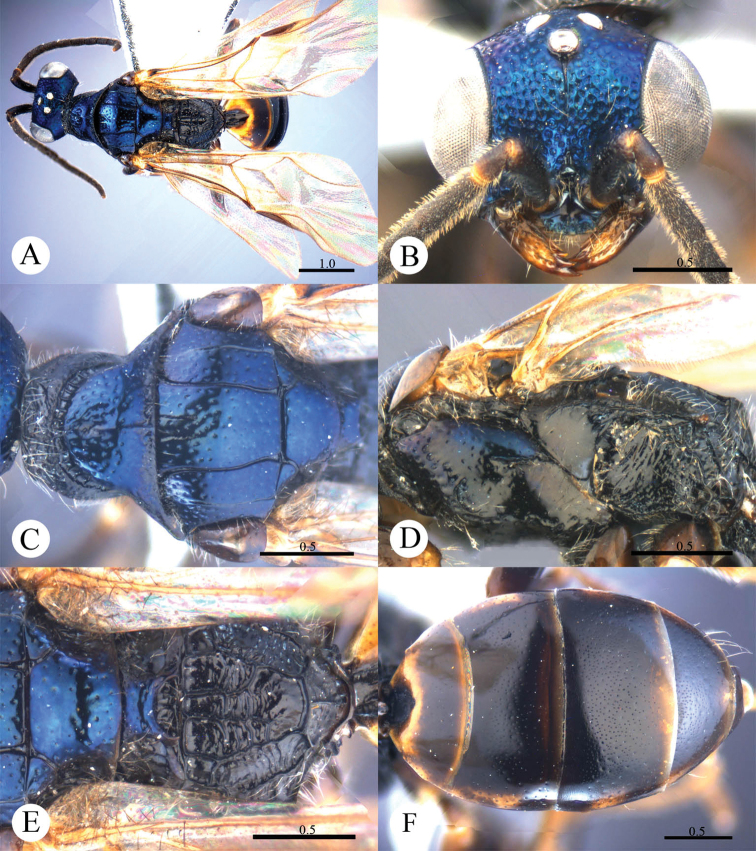
*Cleptes sinensis* sp. n., holotype, male. **A** Habitus dorsal **B** Head anterior **C** Pronotum and mesoscutum dorsal **D** Mesopleuron and metapleuron lateral **E** Mesoscutellum, metanotum and propodeum dorsal **F** Metasoma dorsal. Scale bars in mm.

*Male*. Holotype. Body length 6.2 mm (Plate 11A). Forewing length 4.9 mm. HW: HH: HL = 13: 10: 7.5. POL: OOL: OCL = 8.5: 15.5: 15. MS = 0.6 MOD. Width of clypeal lower margin = 1.3 ASD. L/W of Ped, F-I, F-II, and F-III are 2.1, 3.2, 2.1, and 2.1, respectively.

*Head*. Face with dense and coarse punctures (0–0.5 PD). Clypeus with lower margin indistinctly convex medially, without acute teeth at corners. Frontal sulcus complete (Plate 11B). Mandibles mostly polished, with few punctures and three teeth. Ocellar area with sparser punctures (0.5 PD) than those on face. Ocellar triangle isosceles, with post-ocellar sulcus. Vertex with punctures shallower and sparser (0.5–1.0 PD).

*Mesosoma*. Punctures small, shallow and sparse (0.5–1.5 PD) on pronotum, and even sparser on mesonotum and metanotum. Pronotum with a distinct anterior pit row, without posterior pit row (Plate 11C); without longitudinal median sulcus, with shallow depression in the middle of pronotum (Plate 11C). Mesoscutum with notauli complete; parapsidal lines incomplete, 3/4 length of notauli; admedian lines incomplete, 1/3 length of notauli (Plate 11C); axillary trough longitudinally striate. Mesopleuron polished, with short scrobal sulcus and sparse punctures (Plate 11D). Metanotum without anteromedian pit, with a broad fovea along the posterior margin; axillary trough polished (Plate 11E). Metapleuron smooth and polished (Plate 11D). Dorsal surface of propodeum with six longitudinal ridges, with numerous and weak transverse wrinkles. Propodeal angles long and blunt (Plate 11E).

*Metasoma*. T-I nearly impunctate. T-II–T-IV with small and dense punctures. Punctures on T-III denser than those on T-II and T-IV. T-V with posterior margin emarginate medially (Plate 11F).

*Pubescence*. Head on clypeus with short (0.5–1.0 MOD), sparse and white hairs; on vertex nearly without hairs. Metasoma nearly without hairs, only with few on T-IV and T-V laterally.

*Colouration*. Head metallic blue. Mandibles blackish-brown, with testaceous in the middle. Antennae blackish-brown, with pedicels brown. Mesosoma metallic blue, with propodeum, mesopleuron and metapleuron black. Tegulae brown. Legs blackish-brown, with tarsi testaceous. Mesopleuron, metapleuron and propodeum black. Metasoma blackish-brown, with T-I anteriorly and all segments laterally and along the posterior margin testaceous.

*Variation*. Body length 4.3–6.6 mm. Forewing length 3.9–5.3 mm. Vertex metallic blue, with some purple tints. Mesonotum and metanotum metallic blue, with more or less blackish-brown tints. One specimen from Hainan with blackish-brown mesosoma, and indistinct metallic blue reflections. Pronotum with irregular and shallow punctures along the posterior margin. Metasoma with few 1.5–2.0 MOD long and sparse hairs on T-I, T-II, and T-III laterally.

*Female*. Unknown.

#### Distribution.

China (Shaanxi, Zhejiang, Hubei, Hainan, Sichuan).

#### Biology.

Collected from May, July and August at 1344 m to 1632 m.

#### Remarks.

According to Móczár (1997b), *Cleptes sinensis* sp. n. belongs to the *nitidulus* species-group based on the pronotum without posterior pit row and longitudinal median sulcus, and the blackish-brown metasoma.

### Cleptes sjostedti

Hammer, 1950

http://species-id.net/wiki/Cleptes_sjostedti

Plates 12 , 13

Cleptes sjostedti Hammer, 1950: 2; Kimsey and Bohart 1991: 64.
Cleptes pinicola Lin, 1959: 205. Synonymized by Móczár 1998a.
Cleptes sjoestedti (writing in error) Hammer: Móczár 1998a: 340.

#### Material examined.

Holotype ♀ (NMW), “Provins Kiangsu [= Jiangsu]”, “China *Kolthoff*”, “okt”, “Type”, “*Cleptes sjostedti*, ♀, det. Hammer”, “Holotype, ♀, *Cleptes sjostedti* Hammer, P. Rosa vidit 2012”, “NHRS-HEVA, 000001124”. Other material examined: 1 ♀ (SCAU), Hunan, Liuyang City (28°09'N, 113°38'E), 1548 m, 14.VIII.1984, Xin-wang Tong leg., No. SCAU-C0008; 1 ♀ (SCAU), Hunan, Liuyang City, 1959 m, 19.X.1984, Xin-wang Tong leg., No. SCAU-C0009; 1 ♀ (ZJUH), Yunnan, Xiangyun (25°28'19.93"N, 100°33'13.11"E), V.1980, Hai-lin Wang leg., host: Diprionidae, No. 888669; 1 ♀ (ZJUH), Zhejiang, Mt. Mogan (30°35'53.09"N, 119°54'6.15"E), 12.VI.1982, Wei Lin leg., No. 923040; 2 ♀ (ZJUH), Zhejiang, Anji (30°37'47.40"N, 119°40'53.06"E), 1991, Guo-rong Yang leg., host: Diprionidae, No. 916001; 1 ♀ (ZJUH), Guangdong, Xinhui (22°26'52.14"N, 113°2'4.93"E), 25.VIII.1989, Chuan-chuan Lu leg., No. 896511; 4 ♀+1 ♂ (ZJUH), Yunnan, Kunming (24°52'23.65"N, 102°50'0.64"E), 4.VI.1976, Jing-liang Qi leg., host: Diprionidae, No. 771985; 2 ♀+2 ♂ (ZJUH), Anhui, Ningguo (30°37'42.27"N, 118°58'57.58"E), 1991, Zong-ying Wang leg., No. 940436; 1 ♂ (ZJUH), Zhejiang, Gaozhou, Bamen (30°15'N, 120°9'E), 26.V.1984, Jun-hua He leg., host: Diprionidae, No. 844852.

#### Diagnosis.

Posterior pit row of pronotum with two median pits considerably larger than the others. Pronotum with longitudinal median sulcus incomplete, and somewhat foveate. Mesopleuron with strong foveate V-shape loop. Metanotum with a big, deep and triangular anteromedian pit, with two foveae along the posterior margin.

#### Description.

**Plate 12.**
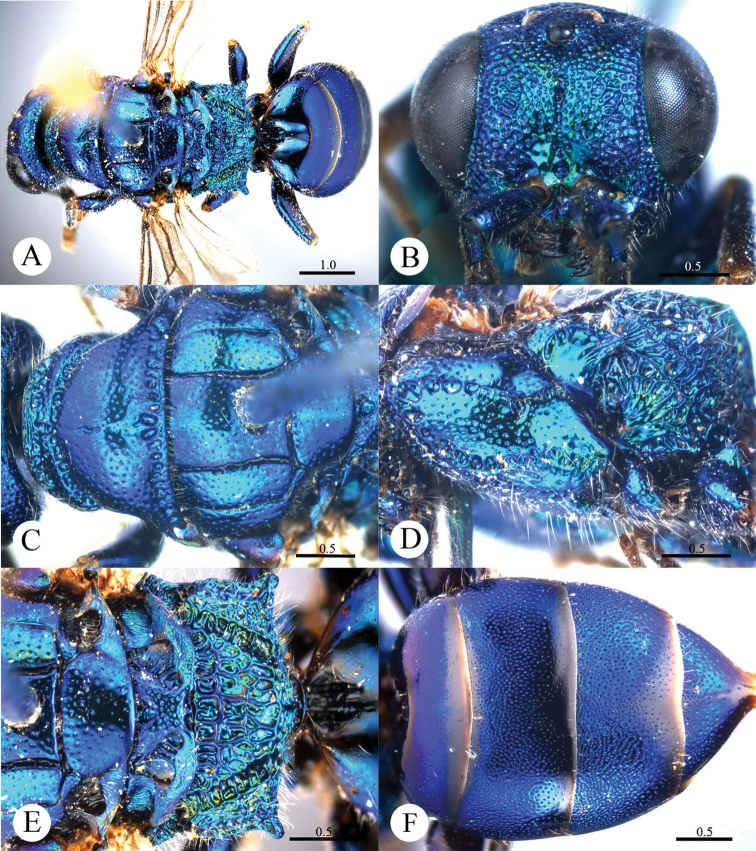
*Cleptes sjostedti* Hammer, 1950, female from Hunan. **A** Habitus dorsal **B** Head anterior **C** Pronotum and mesoscutum dorsal **D** Mesopleuron and metapleuron lateral **E** Mesoscutellum, metanotum and propodeum dorsal **F** Metasoma dorsal. Scale bars in mm.

Redescribed after a female from Hunan (Liuyang City). Body length 8.2 mm (Plate 12A). Forewing length 5.0 mm. HW: HH: HL = 37.5: 30.5: 19. POL: OOL: OCL = 16: 16: 27.5. MS = 1.4 MOD. Width of clypeal lower margin = 1 ASD. L/W of Ped, F-I, F-II, and F-III are 2.0, 2.0, 0.9, and 1.0, respectively.

*Head*. Face with dense and coarse punctures (0–0.5 PD) merging laterally, with big polish area above clypeus. Clypeus with lower margin slightly convex medially, with distinct acute teeth at corners. Frontal sulcus complete, but becoming shallow and indistinct on upper 1/3, after reaching pit before midocellus (Plate 12B). Mandibles with sparse punctures and three teeth. Ocellar area and vertex with shallower and sparser punctures (0.5–1 PD). Ocellar triangle isosceles, without post-ocellar sulcus.

*Mesosoma*. Mesosoma with small and shallow punctures, and gradually sparser from pronotum to metanotum. Pronotum with distinct anterior and posterior pit rows, with two median pits of posterior pit row considerably larger than the others (Plate 12C); longitudinal median sulcus incomplete, and somewhat foveate. Mesoscutum with notauli complete; parapsidal lines nearly complete; admedian lines incomplete, 1/8 length of notauli (Plate 12C); axillary trough longitudinally striate. Mesopleuron with strong foveate V-shape loop (Plate 12D). A transverse foveate sulcus present between mesoscutellum and metanotum (Plate 12E). Metanotum with a big, deep and triangular anteromedian pit, with two foveae along the posterior margin; axillary trough with several oblique wrinkles (Plate 12E). Metapleuron polished, with big and triangular pit on upper part (Plate 12D). Dorsal surface of propodeum rectangular, with width longer than length (L: W = 1: 3), reticulate, with aligned areolae along the anterior and lateral margins. Propodeal angles big and blunt produced obliquely (Plate 12E).

*Metasoma*. T-I with small and sparse punctures. T-II–T-IV with bigger and denser punctures (Plate 12F). Punctures on T-IV double than those on T-II and T-III, with bigger punctures deeply incised. T-IV with apex medially incised.

*Pubescence*. Head with short (0.5–1.0 MOD), sparse and brownish hairs, scattered on clypeus and posterior half of vertex. Metasoma nearly without hairs dorsally, with only few very short (0.5 MOD) hairs laterally.

*Colouration*. Head and mesosoma metallic blue, with purple reflections. Mandibles testaceous, with teeth blackish-brown. Antennae black, with scapes and pedicels metallic blue, ventral sides of F-IV to F-XI testaceous. Tegulae metallic blue, with brown tint. Legs metallic blue, with ventral side of tibiae and tarsi brown. Metasoma metallic bluish-purple.

**Plate 13.**
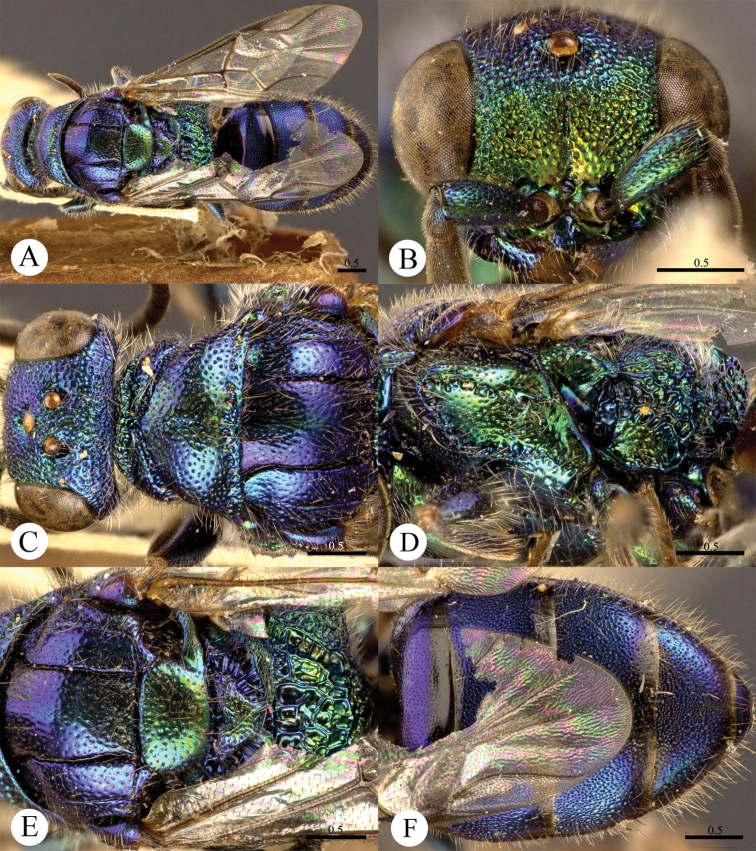
*Cleptes sjostedti* Hammer, 1950, male from Zhejiang. **A** Habitus dorsal **B** Head anterior **C** Head, pronotum and mesoscutum dorsal **D** Mesopleuron and metapleuron lateral **E** Mesoscutum, mesoscutellum, metanotum and propodeum dorsal **F** Metasoma dorsal. Scale bars in mm.

*Male*. Body length 6.1–6.7 mm (Plate 13A). Forewing length 3.6–4.3 mm. Differing from female as follows: pronotum with longitudinal median sulcus deeper (Plate 13C); posterior pit row less distinct, with median pair of pits bigger than the others (Plate 13C); body largely purple with metallic blue reflections (Plate 13B, 13D–F).

*Variation*. *Females*. Body length 5.8–8.2 mm. Forewing length 3.6–5.0 mm. Clypeus almost truncate medially, with distinct acute teeth at corners. Males. Body length 6.1–6.7 mm. Forewing length: 3.6–4.3 mm. Body largely purple with metallic blue reflections.

#### Distribution.

China (Jiangsu, Anhui, Zhejiang, Taiwan, Hunan, Guangdong, Yunnan); Korea.

#### Biology.

Parasitoid of Diprionidae. Collected from May, June, August and October at 1548 m to 1959 m.

#### Remarks.

*Cleptes sjostedti* Hammer belongs to the *fudzi* species-group (Móczár 1998a). The correct writing of the name is *sjostedti* and not *sjoestedti* as reported in Móczár (1998a). The species was dedicated to Yngve Sjöstedt, former director of the Naturhistoriska Riksmuseum in Stockholm. According to the ICZN (Art. 32.5.2.1) only in the case of a German name would the correct form be *sjoestedti*.

### Cleptes taiwanus

Tsuneki, 1982

http://species-id.net/wiki/Cleptes_taiwanus

Cleptes taiwanus Tsuneki, 1982: 2; Móczár 2000a: 329.

#### Material examined.

None.

#### Diagnosis.

Body mostly metallic greenish-blue, with propodeum, part of mesonotum and partly T-I–IV purple. Tegulae metallic greenish-blue basally, brown apically with purple tint. Lower margin of clypeus with acute teeth at corners. Ocellar triangle isosceles. Parapsidal lines complete. Metanotum with an anteromedian pit and a broad fovea along the posterior margin (Tsuneki 1982; Móczár 2000a).

#### Distribution.

Oriental part of China (Taiwan).

#### Biology.

Collected in August.

#### Taxonomic remarks.

*Cleptes taiwanus* Tsuneki belongs to *asianus* species-group (Móczár 2000a).

### Cleptes tibetensis sp. n.

http://zoobank.org/5C856F83-1F7D-4752-A0E0-D19E3D51659C

http://species-id.net/wiki/Cleptes_tibetensis

Plate 14

#### Material examined.

Holotype ♂ (SCAU), Tibet, Pailongxiang, Daxiagu (30°1'10.56"N, 94°59'49.92"E), 2054 m, 15.VI.2009, Mei-cai Wei leg., No. SCAU-C0003.

#### Diagnosis.

*Cleptes tibetensis* sp. n. is similar to *Cleptes crassiceps* Tsuneki, *Cleptes metallicorpus* Ha, Lee & Kim, and *Cleptes villosus* sp. n. based on the posterior pit row on pronotum, V-shape loop on mesopleuron, and short propodeal angles. However, it can be distinguished by the combination of the following characteristics: metallic bluish-purple metasoma (metasoma black, only partly with metallic reflections in *Cleptes crassiceps*); absence of frontal sulcus (present in *Cleptes crassiceps*, *Cleptes metallicorpus* and *Cleptes villosus* sp. n.); lower margin of clypeus with acute teeth at corners (absent in *Cleptes crassiceps* and *Cleptes villosus* sp. n.); mandibles without striatopunctures (with striatopunctures in *Cleptes metallicorpus*); metanotum with transverse depression anteriorly (absent in *Cleptes crassiceps*, *Cleptes metallicorpus*, and *Cleptes villosus* sp. n.); metanotum with a broad fovea along the posterior margin (with two foveae along the posterior margin in *Cleptes metallicorpus*).

#### Description.

**Plate 14.**
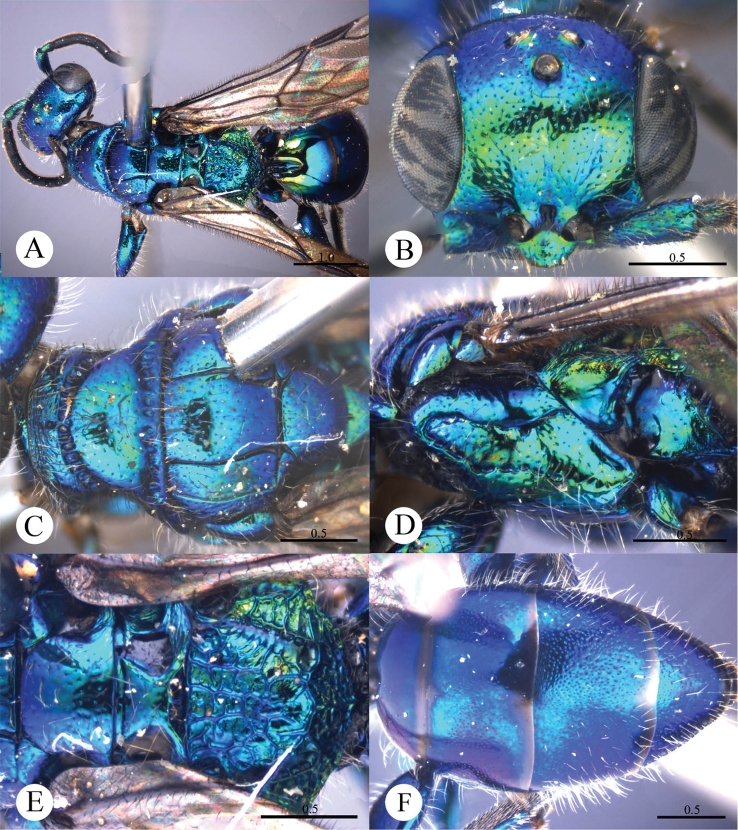
*Cleptes tibetensis* sp. n.,holotype,male**. A** Habitus dorsal **B** Head anterior **C** Pronotum and mesoscutum dorsal **D** Mesopleuron and metapleuron lateral **E** Mesoscutellum, metanotum and propodeum dorsal **F** Metasoma dorsal. Scale bars in mm.

*Male*. Holotype. Body length 6.7 mm (Plate 14A). Forewing length 5.6 mm. HW: HH: HL = 18.8: 12.5: 10.5. POL: OOL: OCL = 5: 9.8: 12.3. MS = 0.9 MOD. Width of clypeal lower margin = 1 ASD. L/W of Ped, F-I, F-II, and F-III are 1.5, 2.1, 1.7, and 1.7, respectively.

*Head*. Face, ocellar area and vertex with small and sparse punctures (1–5 PD). Clypeus with lower margin slightly convex medially, with acute teeth at corners. Frontal sulcus absent (Plate 14B). Mandibles with sparse punctures. Ocellar triangle equilateral, without post-ocellar sulcus.

*Mesosoma*. Mesosoma with punctures similar to those on vertex. Pronotum with distinct anterior and posterior pit rows (Plate 14C); without longitudinal median sulcus (Plate 14C). Mesoscutum with notauli complete; parapsidal lines nearly complete; admedian lines incomplete, 1/6 length of notauli (Plate 14C); axillary trough smooth. Mesopleuron with distinct V-shape loop (Plate 14D). Metanotum with transverse depression anteriorly, with a small and oval anteromedian pit; with a broad fovea along the posterior margin; axillary trough smooth and polished (Plate 14E). Metapleuron polished, with some indistinct transverse wrinkles on upper part (Plate 14D). Dorsal surface of propodeum with irregular ridges; lateral margins parallel. Propodeal angles short and stumpy (Plate 14E).

*Metasoma*. T-I and T-V impunctate. T-II–T-IV with dense punctures (Plate 14F). Punctures on T-III denser than those on T-II and T-IV.

*Pubescence*. Head with long (1.0–1.3 MOD), sparse and whitish hairs. Metasoma laterally and on T-III and T-IV also dorsally with long (1.5–2.0 MOD) and whitish hairs.

*Colouration*. Face and clypeus yellowish-green. Vertex metallic green, with purple tints. Mandibles metallic green, with teeth blackish-brown. Antennae blackish-brown, with scapes and pedicels metallic green. Mesosoma mostly metallic green, with some purple tints. Tegulae metallic green. Legs metallic green, with tarsi testaceous. Metasoma metallic bluish-purple, with T-I yellowish-green tints anteriorly, with T-I bluish-green tints laterally; T-V black.

*Female*. Unknown.

#### Distribution.

China (Tibet).

#### Biology.

Collected in June at 2054 m.

#### Etymology.

The species is named after the type locality.

#### Remarks.

According to Móczár (2000a), *Cleptes tibetensis* sp. n. belongs to the *asianus* species-group based on the two distinct pit rows on pronotum, longitudinal median sulcus absent, and mesopleuron with V-shape loop.

### Cleptes townesi

Kimsey, 1987

http://species-id.net/wiki/Cleptes_townesi

Plate 15

Cleptes townesi Kimsey, 1987: 58; Kimsey and Bohart 1991: 64; Móczár 2000a: 330.

#### Material examined.

2 ♂ (SCAU), Zhejiang, Hangzhou (30°16'32.72"N, 120°9'16.38"E), 21.V.1981, Yun Ma leg., No. 810679; 1 ♂ (SCAU), Fujian, Chong’an, Mt. Wuyi, Jiuqu (27°39'12.88"N, 117°56'24.43"E), 27.IV.1984, Xiu-fu Zhao leg., No. 20007514.

#### Diagnosis.

Metallic blue reflection restricted to face. Pronotum with anterior pit row distinct and posterior pit row absent. Mesopleuron polished, with sparse, shallow and aligned punctures on anterior half, with short scrobal sulcus. Metanotum without anteromedian pit, with a broad fovea along the posterior margin. T-V with posterior margin emarginate medially.

#### Description.

**Plate 15.**
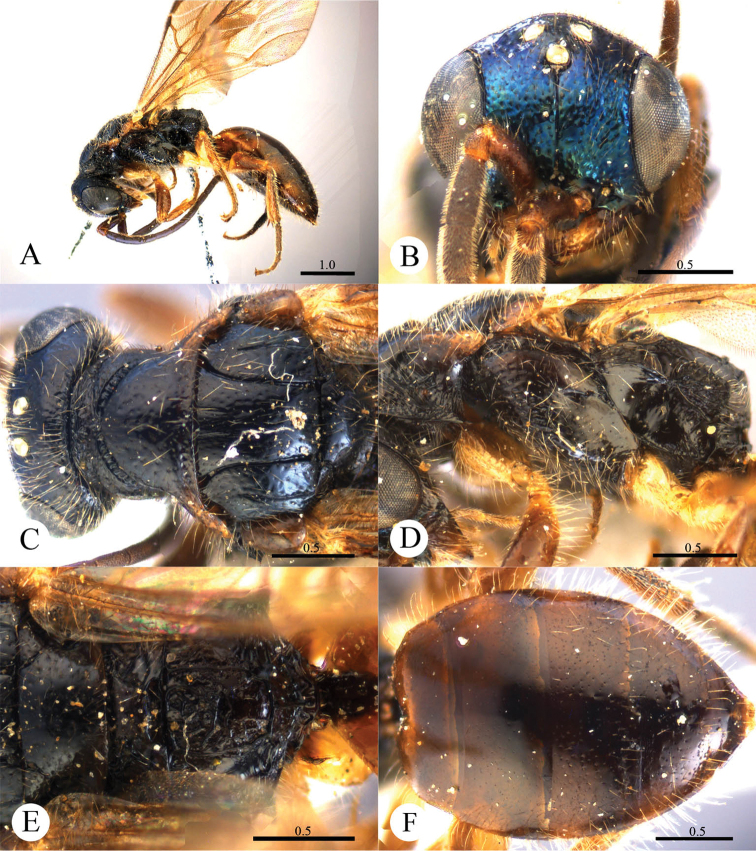
*Cleptes townesi* Kimsey, 1987, male from Zhejiang. **A** Habitus lateral **B** Head anterior **C** Head, pronotum and mesoscutum dorsal **D** Mesopleuron and metapleuron lateral **E** Mesoscutellum, metanotum and propodeum dorsal **F** Metasoma dorsal. Scale bars in mm.

Redescribed after a male from Zhejiang. Body length 5.6 mm (Plate 15A). Forewing length 4.6 mm. HW: HH: HL = 13.5: 10: 5.5. POL: OOL: OCL = 9.5: 16.5: 15.5. MS = 0.6 MOD. Width of clypeal lower margin = 1.7 ASD. L/W of Ped, F-I, F-II, and F-III are 1.2, 2.6, 2.0, and 2.1, respectively.

*Head*. Face and vertex with small and sparse punctures (0.5–1.0 PD). Clypeus with lower margin truncate, without acute teeth at corners. Frontal sulcus complete (Plate 15B). Mandibles mostly polished, with few punctures and four teeth. Ocellar area with punctures similar to those on vertex but denser (0.5 PD). Ocellar triangles isosceles, almost equilateral, bulging in frontal view, without post-ocellar sulcus.

*Mesosoma*. Pronotum with shallow and sparse punctures (1–2 PD). Pronotum with anterior pit row distinct and posterior pit row absent (Plate 15C); without longitudinal median sulcus (Plate 15C). Mesonotum and metanotum with smaller punctures than those on pronotum. Mesoscutum with notauli complete; parapsidal lines incomplete, 3/4 length of notauli; admedian lines incomplete, 1/4 length of notauli (Plate 15C); axillary trough weakly reticulate. Mesopleuron polished, with sparse, shallow and aligned punctures on anterior half, with scrobal sulcus short (Plate 15D). A foveate transverse sulcus present between mesoscutellum and metanotum (Plate 15E). Metanotum without anteromedian pit, with a broad fovea along the posterior margin; axillary trough with few weak and oblique transverse wrinkles (Plate 15E). Metapleuron mostly smooth and polished (Plate 15D). Dorsal surface of propodeum irregularly reticulate; lateral margins parallel. Propodeal angles short and stumpy, slightly divergent (Plate 15E).

*Metasoma*. T-I and T-V nearly impunctate. T-II with sparse punctures. T-III and T-IV with dense punctures (Plate 15F). T-V with posterior margin emarginate medially.

*Pubescence*. Head on vertex with long (1.0–1.5 MOD), sparse and brownish hairs. Metasoma on T-I and T-II laterally, on T-III to T-V laterally and dorsally with long (1.0–1.5 MOD) and brownish hairs.

*Colouration*. Metallic blue reflections restricted to face. Vertex and mesosoma black, without metallic reflections. Mandibles brown, with teeth testaceous. Antennae blackish-brown, with scapes and pedicels brown. Tegulae brown. Legs brown, with coxae and trochanters testaceous. Metasoma brown.

*Variation*. Body length 5.6–6.7 mm. Forewing length 4.4–5.1 mm.

*Female*. Unknown.

#### Distribution.

Oriental part of China (Zhejiang, Fujian, Taiwan).

#### Biology.

Collected in April and May.

#### Remarks.

*Cleptes townesi* Kimsey belongs to the *townesi* species-group (Móczár 2000a).

### Cleptes villosus sp. n.

http://zoobank.org/F4FC46FB-2B2F-4195-BA3A-B0FB02A5BE56

http://species-id.net/wiki/Cleptes_villosus

Plate 16

#### Material examined.

Holotype ♂ (SCAU), Guizhou, Suiyang, Kuankuoshui National Nature Reserve (28°1'23.16"N, 107°7'45.29"E), 8.VI.2010, Jie Zeng leg., No. SCAU-C0006. Paratype: 1 ♂ (ZJUH), Guizhou, Daozhen, Dashahe, Xiannvdong (29°2'38.19"N, 107°30'31.13"E), 644 m, 24.VIII.2004, Shu-jun Wei leg., No. 20047405.

#### Diagnosis.

*Cleptes villosus* sp. n. is similar to *Cleptes metallicorpus* Ha, Lee & Kim, *Cleptes taiwanus* Tsuneki, *Cleptes thaiensis* Tsuneki and *Cleptes tibetensis* sp. n. based on the mesopleuron with V-shape loop, metanotum with an anteromedian pit and short propodeal angles. However, it can be separated by the combination of the following characteristics: the metallic bluish-green body (purple with greenish golden or blue tints in *Cleptes thaiensis*); lower margin of clypeus without acute teeth (present in *Cleptes metallicorpus*, *Cleptes taiwanus* and *Cleptes tibetensis* sp. n.); mandibles without striatopunctures (with distinct striatopunctures in *Cleptes metallicorpus*); ocellar triangle equilateral, without post-ocellar sulcus (ocellar triangle isosceles in *Cleptes metallicorpus* and *Cleptes taiwanus*, with post-ocellar sulcus in *Cleptes taiwanus*); and long, dense hairs on head and mesosoma.

#### Description.

**Plate 16.**
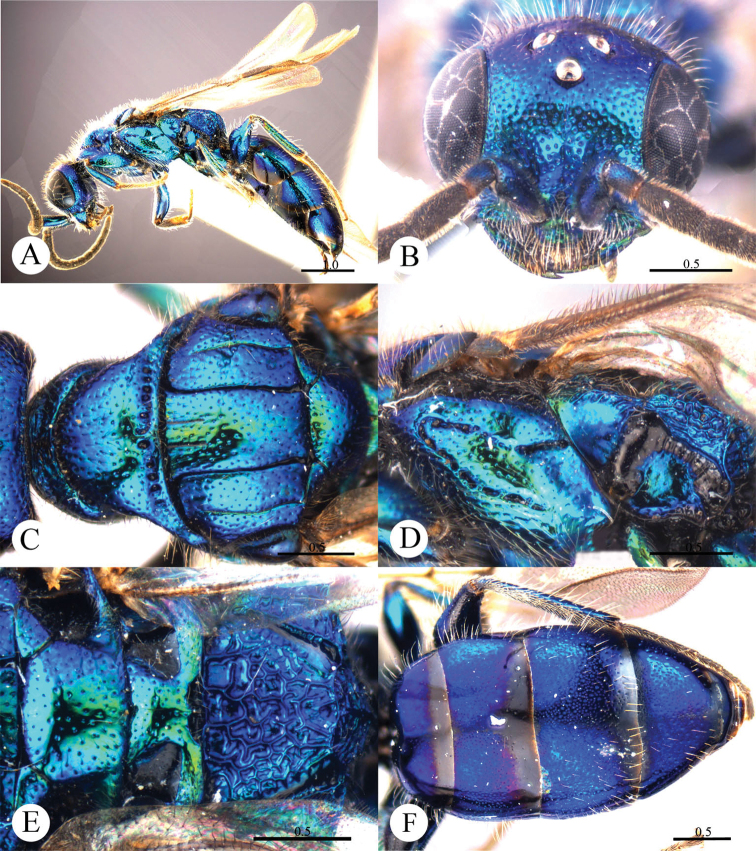
*Cleptes villosus* sp. n., holotype, male. **A** Habitus lateral **B** Head anterior **C** Pronotum and mesoscutum dorsal **D** Mesopleuron and metapleuron lateral **E** Mesoscutellum, metanotum and propodeum dorsal **F** Metasoma dorsal. Scale bars in mm.

*Male*. Holotype. Body length 6.4 mm (Plate 16A). Forewing length 5.0 mm. HW: HH: HL = 21: 13: 8.8. POL: OOL: OCL = 5.5: 7.8: 12. MS = 1 MOD. Width of clypeal lower margin = 1 ASD. L/W of Ped, F-I, F-II, and F-III are 1.6, 1.8, 1.7, and 1.4, respectively.

*Head*. Face and ocellar area with small, shallow and dense punctures (0.5–1.0 PD). Clypeus with lower margin truncate, without acute teeth at corners. Frontal sulcus complete but weak (Plate 16B). Mandibles with sparse punctures and four teeth. Ocellar triangle equilateral, without post-ocellar sulcus. Vertex with shallower and sparser punctures (1.0–1.5 PD).

*Mesosoma*. Mesosoma with small and sparse punctures (1.5–3.0 PD). Pronotum with distinct anterior and posterior pit rows (Plate 16C); without longitudinal median sulcus (Plate 16C). Mesoscutum with notauli complete; parapsidal lines nearly complete; admedian lines incomplete, 1/3 length of notauli (Plate 16C); axillary trough smooth. Mesopleuron with foveate V-shape loop (Plate 16D). Metanotum with a small and oval anteromedian pit, with two medially fused foveae along the posterior margin; axillary trough smooth (Plate 16E). Metapleuron mostly smooth and polished (Plate 16D). Dorsal surface of propodeum irregularly reticulate. Propodeal angles short and blunt (Plate 16E).

*Metasoma*. Posterior margin of each segment of metasoma impunctate. T-I and T-V nearly impunctate. T-II with small and dense punctures, only scattered on anterior 2/3 (Plate 16F). T-III with punctures twice as dense as on T-II, with very small dots in intervals between the larger punctures. T-IV with punctures on slightly sparser than on T-III.

*Pubescence*. Head with long (1.5 MOD), dense and white hairs. Mesosoma with long (1.5–2.0 MOD), dense and whitish hairs. Metasoma laterally on T-I and T-II and on T-III with long (1.5–2.0 MOD), sparse and white hairs; T-IV with short (0.5–0.8 MOD) and sparse hairs; T-V with only few short (0.5–0.8 MOD) hairs.

*Colouration*. Body mostly metallic bluish-green, with vertex mostly purple and with metallic blue tints. Mandibles metallic bluish-green, with teeth blackish-brown. Antennae black, with scapes and pedicels metallic bluish-green. Tegulae metallic bluish-green. Legs metallic bluish-green, with tibiae and tarsi testaceous. Metasoma purple, with metallic blue tints, posterior margin of each segment black, T-V blackish-blue.

*Variation*. Paratype.Body length 5.7 mm. Forewing length 4.7 mm. Clypeus covered with sparser and shorter setae.

*Female*. Unknown.

#### Distribution.

Oriental part of China (Guizhou).

#### Biology.

Collected in June and August.

#### Etymology.

The specific name refers to the whitish, dense relatively long hairs on the head and mesosoma.

#### Remarks.

According to Móczár (2000a), *Cleptes villosus* sp. n. belongs to the *asianus* species-group based on the two pit rows on pronotum, longitudinal median sulcus absent, and mesopleuron with V-shape loop.

## Discussion

We consider the occurrence of *Cleptes nitidulus* in China reported by Tsuneki (1959) as a misidentification (Table 1). Tsuneki (1959) examined only one male *Cleptes nitidulus* from Manchuria (Northeast China), but its description does not match the current interpretation of the species based on the holotype or to any other *Cleptes nitidulus* described by other authors (Móczár 1997b; Rosa 2006). Moreover, an earlier report of *Cleptes nitidulus* in the East Palaearctic (Uchida 1926) was also considered debatable by Ha et al. (2011). Therefore, *Cleptes nitidulus* is temporarily excluded from the checklist of the Chinese *Cleptes* until a new examination of Tsuneki’s specimen.

Similarly, Sheng et al. (1998) reported *Cleptes semiauratus* as a new record to China. After examining the specimens, it evidently belongs to an undescribed species (*Cleptes shengi* sp. n.). Despite of other characteristics, *Cleptes shengi* sp. n. can be quickly distinguished from *Cleptes semiauratus* by the dark colouration, while the latter usually with the head and pronotum flame red, mesonotum and metanotum golden red or golden green (Móczár 2001; Rosa 2006).

Interestingly, the *Cleptes* species in China show a convergence to green, blue and black in all studied species, while those in the Western Palaearctic show various metallic and non-metallic colours (such as green, blue, orange, red, golden tinge, and so on) with different combinations. The reason of this phenomenon needs further research.

## Supplementary Material

XML Treatment for Cleptes

XML Treatment for Cleptes asianus

XML Treatment for Cleptes albonotatus

XML Treatment for Cleptes eburnecoxis

XML Treatment for Cleptes flavolineatus

XML Treatment for Cleptes helanshanus

XML Treatment for Cleptes mandsuricus

XML Treatment for Cleptes mareki

XML Treatment for Cleptes metallicorpus

XML Treatment for Cleptes niger

XML Treatment for Cleptes seoulensis

XML Treatment for Cleptes shengi

XML Treatment for Cleptes sinensis

XML Treatment for Cleptes sjostedti

XML Treatment for Cleptes taiwanus

XML Treatment for Cleptes tibetensis

XML Treatment for Cleptes townesi

XML Treatment for Cleptes villosus
